# Oxidative denaturation of Cu/Zn‐superoxide dismutase associated with neurodegenerative diseases

**DOI:** 10.1002/pro.70339

**Published:** 2025-10-16

**Authors:** Moeno Yoshida, Norifumi Muraki, Michiko Tajiri, Kowit Hengphasatporn, Kaori Sue, Takehiro Kubo, Satoko Akashi, Yasuteru Shigeta, Taiho Kambe, Yoshiaki Furukawa

**Affiliations:** ^1^ Department of Chemistry Keio University Yokohama Japan; ^2^ Graduate School of Medical Life Science Yokohama City University Yokohama Japan; ^3^ Center for Computational Sciences University of Tsukuba Tsukuba Japan; ^4^ Graduate School of Biostudies Kyoto University Kyoto Japan

**Keywords:** neurodegenerative diseases, protein denaturation, superoxide dismutase, thiol oxidation

## Abstract

Misfolding of mutant Cu/Zn‐superoxide dismutase (SOD1) is a well‐established pathological feature of familial amyotrophic lateral sclerosis (ALS). While amino acid substitutions in mutant SOD1 destabilize its structure and promote misfolding, oxidation has also been implicated in the pathological alterations of wild‐type SOD1, particularly in neurodegenerative diseases including sporadic ALS. However, the impact of oxidation on SOD1 folding remains to be fully elucidated. Here, we demonstrate that Cys111 is primarily oxidized to sulfonic acid upon exposure of apo‐SOD1 to hydrogen peroxide, as confirmed by the quantitation of thiol groups and mass spectrometry. Molecular dynamics simulations showed that sulfonylation of Cys111 disrupts the dimer interface and promotes monomerization. This monomeric form then facilitates the subsequent oxidation of buried Cys6, leading to structural disruption, as evidenced by circular dichroism spectroscopy and loss of thiol groups. SOD1 denaturation triggered by Cys111 oxidation became evident when zinc binding was impaired due to pathological mutations and/or under zinc‐deficient conditions. Given that increased oxidative stress is frequently associated with many neurodegenerative diseases, modulating Cys111 oxidation may offer a potential strategy for maintaining SOD1 structural stability and preventing its pathological misfolding.

## 1 INTRODUCTION

Mutations in the gene encoding Cu/Zn‐superoxide dismutase (SOD1) are well known to cause familial amyotrophic lateral sclerosis (ALS) (Rosen et al., 1993). A pathological hallmark of familial ALS with *SOD1* mutations is the abnormal accumulation of misfolded mutant SOD1 proteins in motoneurons (Bruijn et al., 1998). Even in the absence of mutations, wild‐type SOD1 has also been reported to undergo conformational alterations in sporadic ALS, although this remains controversial (Furukawa & Tokuda, 2020), suggesting that factors other than pathogenic amino acid substitutions may significantly influence SOD1 conformation. Notably, increased oxidative stress is frequently associated with many neurodegenerative diseases (Teleanu et al., 2022), and an oxidized form of wild‐type SOD1 has been detected in several, including sporadic ALS (Abdeen et al., 2025; Choi et al., 2005; Guareschi et al., 2012; Tokuda et al., 2019). Thus, oxidative modifications may serve as a pathological factor in triggering SOD1 misfolding.

**FIGURE 1:**
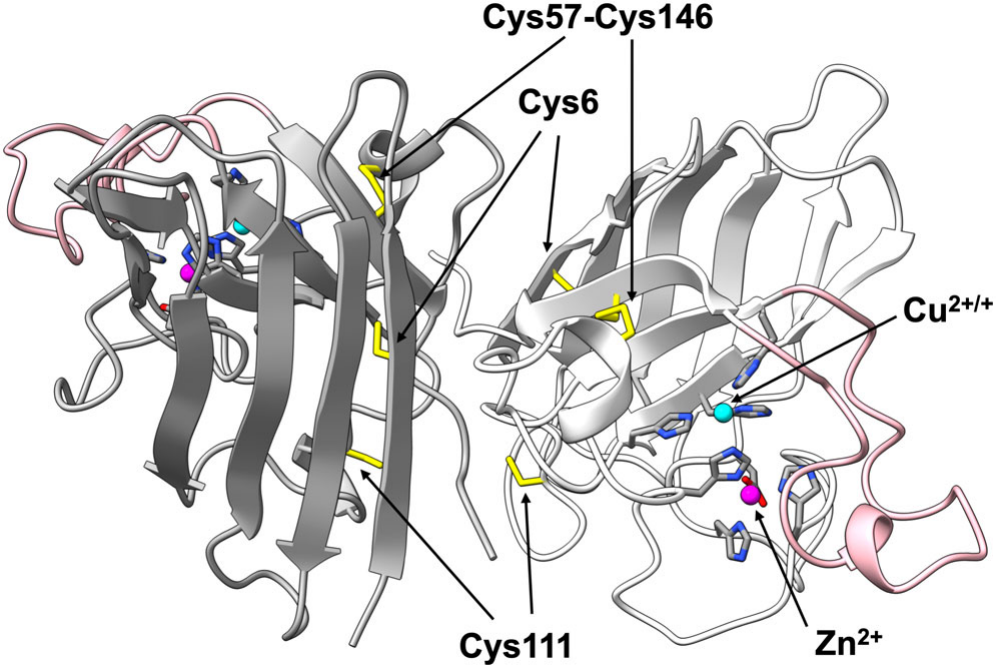
Crystal structure of human SOD1 (PDB ID: 1HL5). The structure of human SOD1 is shown as a cartoon model. One subunit (right) is colored white, and the other subunit (left) is shown in gray. A copper and zinc ion bound to each subunit are depicted as a cyan and magenta sphere, respectively, with their coordinating residues shown as stick models. All cysteine residues are also shown in yellow stick representation. The electrostatic loop (Glu121–Ser142) is highlighted in pink.

SOD1 is a homodimeric protein, with each subunit binding a copper and zinc ion and forming a conserved intramolecular disulfide bond (Figure 1) (McCord & Fridovich, 1969). At the copper site, SOD1 catalyzes the disproportionation of superoxide to molecular oxygen and hydrogen peroxide (H_2_O_2_); notably, the generated H_2_O_2_ could act as an oxidant for SOD1 itself, potentially influencing its structural stability and function. In the holo form of SOD1 (SOD1 with a copper and zinc ion), metal‐ligating His residues are oxidized upon H_2_O_2_ exposure in vitro, facilitating copper and/or zinc release (Kurahashi et al., 2001; Mulligan et al., 2012; Sato et al., 1992). The liberated copper ion then reacts with H_2_O_2_ to generate hydroxyl radicals, which nonspecifically degrade SOD1 (Ookawara et al., 1992). The oxidized SOD1 has also been proposed to form granular aggregates in the presence of the metal chelator EDTA (Mulligan et al., 2012). Furthermore, in the presence of bicarbonate, the carbonate radicals are generated at the copper site upon reaction with H_2_O_2_ and oxidize the solvent‐exposed Trp32 (Zhang et al., 2003). This results in the formation of a covalent dimer crosslinked by a ditryptophan bond, promoting amorphous aggregation (Coelho et al., 2014). Whether aggregates in neurodegenerative disease patients contain SOD1 with oxidized His/Trp remains to be determined; however, SOD1 is thought to be damaged under increased oxidative stress.

While the copper ion in SOD1 can generate highly reactive species such as hydroxyl and carbonate radicals, metal‐deficient SOD1, often implicated in misfolding (Sheng et al., 2014), is also oxidized by H_2_O_2_ independent of the copper ion, with the thiol (–SH) group of Cys111 preferentially oxidized to sulfenic (–SOH) and sulfonic (–SO_3_H) acids. We previously reported that Cys111 in the apo form of SOD1 undergoes sulfenylation upon exposure to H_2_O_2_ in relatively low concentrations (~60 μM) and that the sulfenylated Cys111 further forms an additional intramolecular disulfide bond with Cys6 (Anzai et al., 2020). Such an aberrant SOD1 species with two intramolecular disulfide bonds exhibits a high propensity for aggregation and significant cytotoxicity (Anzai et al., 2020). Notably, SOD1 with sulfenylated Cys111 has been detected in the cerebrospinal fluid of a subset of sporadic ALS patients without *SOD1* mutations (Xu et al., 2018).

At relatively high concentrations of H_2_O_2_ (~10 mM), the sulfonylation at Cys111 in SOD1 occurs and is characterized by its reduced electrophoretic mobility in SDS‐PAGE (Fujiwara et al., 2007). Mass spectrometric analyses of the detergent‐insoluble fractions from the spinal cords of end‐stage ALS‐model mice expressing human SOD1 variants detected primarily full‐length, unmodified SOD1, with no evidence of oxidized forms (Shaw et al., 2008). This observation has led to the suggestion that oxidized SOD1 may not be incorporated into mature aggregates. Using an antibody specific to SOD1 sulfonylated at Cys111, however, this sulfonylated form was detected in pathological inclusions within the neuropil and neuronal cytoplasm (Fujiwara et al., 2007). Notably, staining was also observed at the rim of neuropil vacuoles, likely corresponding to extracellular spaces formed during neurodegeneration (Fujiwara et al., 2007). Although such inclusions in ALS patients have not been examined for oxidized SOD1, sulfonylated SOD1 has been detected in the cerebrospinal fluid of patients with several neurodegenerative diseases, including sporadic ALS (Tokuda et al., 2019). These findings suggest that sulfonylated SOD1, while absent from mature detergent‐insoluble aggregates, may transiently accumulate in intra‐ or extracellular compartments and contribute to SOD1 toxicity. Indeed, wild‐type SOD1 sulfonylated at Cys111 exhibits toxicity comparable to SOD1 with ALS‐causing mutations; for example, sulfonylated SOD1 was shown to inhibit kinesin‐based fast axonal transport in isolated squid axoplasm (Bosco et al., 2010) and reduce the survival of cultured motor neurons from mouse spinal cords (Ezzi et al., 2007). Furthermore, sulfonylated SOD1 exposes the epitope recognized by the monoclonal antibody C4F6, which specifically detects misfolded SOD1 (Rotunno et al., 2014), suggesting that Cys111 sulfonylation induces SOD1 misfolding.

Several studies have investigated the conformational properties of SOD1 oxidized at high concentrations of H_2_O_2_, reporting increased affinity for copper‐saturated chelating Sepharose resins (Kishigami et al., 2010) and exposure of the β6/β7‐loop region, likely due to disruption of the allosteric network of intramolecular contacts by sulfonylation at Cys111 (Bakavayev et al., 2021). Nonetheless, the mechanistic link between Cys111 oxidation and SOD1 misfolding remains poorly understood. Here, we demonstrate that Cys111 sulfonylation primarily promotes SOD1 monomerization, which subsequently facilitates the oxidation of Cys6. Given its role in stabilizing the hydrophobic core, oxidation of Cys6 increases hydrophilicity and destabilizes the core, ultimately leading to structural denaturation. This sequential oxidation process provides new insights into how oxidative stress may drive SOD1 misfolding and destabilization, potentially contributing to its pathological role in neurodegenerative diseases.

## 2 RESULTS

### 2.1 SOD1 loses structural integrity upon oxidation with H_2_O_2_

**FIGURE 2:**
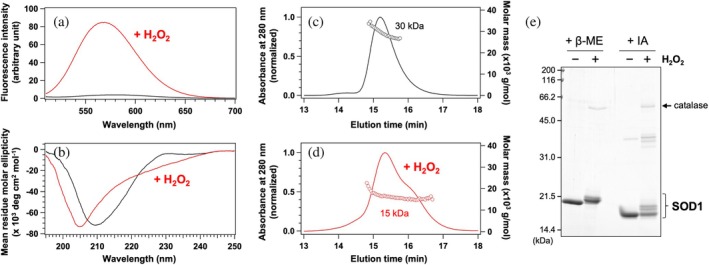
Apo‐SOD1 forms denatured monomers upon oxidation (a) Fluorescence spectra of 10× SYPRO™ Orange with 20 μM apo‐SOD1 (black) before and (red) after treatment with 10 mM H_2_O_2_. (b) CD spectra of 20 μM apo‐SOD1 in 5 mM MOPS/100 mM NaCl (pH 7.0) (black) before and (red) after treatment with 10 mM H_2_O_2_. (c, d) Gel filtration chromatograms of 20 μM apo‐SOD1 in the MN buffer (c) before and (d) after treatment with 10 mM H_2_O_2_, monitored at 280 nm and normalized for comparison (solid lines, left axis), along with molecular masses estimated by MALS (circles, right axis). (e) SDS‐PAGE analysis of apo‐SOD1 before (−) and after (+) treatment with 10 mM H_2_O_2_. Residual H_2_O_2_ was removed by subsequent catalase treatment, which appears as an additional band on the gel (indicated by an arrow). Samples were analyzed under reducing conditions with β‐mercaptoethanol (+β‐ME) or under non‐reducing conditions following modification with iodoacetamide to protect free thiols (+IA).

To investigate structural effects of H_2_O_2_‐induced oxidation on SOD1, apo‐SOD1 (SOD1 lacking a copper and zinc ion but retaining the conserved intramolecular disulfide bond) was prepared at a concentration of 20 μM and incubated with 10 mM H_2_O_2_. Following incubation at 37°C for 24 h, the oxidized SOD1 exhibited significant fluorescence upon binding to SYPRO™ Orange, a dye that fluoresces in hydrophobic environments, while apo‐SOD1 before the oxidation showed minimal fluorescence (Figure 2a). Thus, the oxidation by H_2_O_2_ is considered to cause the exposure of hydrophobic regions within the protein interior, which are typically buried in the native structure, thereby leading to the denaturation of SOD1.

The denaturation of SOD1 upon incubation with H_2_O_2_ was further investigated using CD spectroscopy. The CD spectrum of apo‐SOD1 exhibited a characteristic negative trough at 210 nm (Figure 2b), consistent with previous reports (Anzai et al., 2017). In contrast, H_2_O_2_‐oxidized apo‐SOD1 displayed a markedly different spectrum, featuring a negative trough at 205 nm and a more gradual slope around 225 nm compared with the state before oxidation (Figure 2b). These results indicate that H_2_O_2_ oxidation leads to substantial denaturation at the secondary structural level.

Apo‐SOD1 is a homodimer, with each subunit having a molecular weight of 16 kDa. This was confirmed by a single elution peak observed in the gel filtration chromatogram, during which multi‐angle light scattering (MALS) analysis was performed (Figure 2c). The MALS analysis indicated a molecular weight of 30 kDa for the eluted protein, consistent with the dimeric state of apo‐SOD1. In contrast, H_2_O_2_‐oxidized SOD1 eluted at a similar retention time to that of apo‐SOD1 but exhibited a shoulder at a later elution time (Figure 2d). Nonetheless, MALS analysis indicated molecular weights of 17 and 15 kDa for the species eluted at 15.3 and 16.2 min, respectively, suggesting that H_2_O_2_‐oxidized SOD1 existed in a monomeric state. Given its elution time being comparable to that of dimeric apo‐SOD1, the monomeric H_2_O_2_‐oxidized SOD1 likely adopts a more extended conformation than apo‐SOD1.

Proteins can undergo covalent crosslinking upon oxidation, forming bonds such as disulfide and ditryptophan linkages, which may contribute to denaturation. After blocking free thiols in SOD1 with iodoacetamide, SDS‐PAGE analysis under non‐reducing conditions revealed additional minor bands with slightly reduced mobilities at the monomer position following H_2_O_2_ treatment (Figure 2e, +IA). Although their origin remains uncertain, covalently crosslinked dimeric or higher‐order oligomeric species were only faintly detectable in apo‐SOD1 even after oxidation. Under reducing conditions (Figure 2e, +β‐ME), furthermore, a slight decrease in electrophoretic mobility was observed upon oxidation, consistent with the conversion of Cys111 to sulfonic acid as described in the Introduction. Taken together, these results indicate that covalent crosslinking is not a major contributor to SOD1 denaturation upon H_2_O_2_ oxidation.

Given that oxidized SOD1 has been reported to exhibit cytotoxicity in certain neuronal models (Bosco et al., 2010; Ezzi et al., 2007), we tested its effect on the viability of SH‐SY5Y cells; however, exposure to oxidized apo‐SOD1 at concentrations up to 20 μM did not significantly affect cell viability (Figure S1), suggesting that its toxicity may be cell type dependent or require additional structural modifications. Nonetheless, our results here indicate that oxidation with H_2_O_2_ leads to the loss of structural integrity in apo‐SOD1, characterized by altered secondary structure and monomerization.

### 2.2 H_2_O_2_ oxidizes thiol groups of Cys residues in apo‐SOD1

**FIGURE 3:**
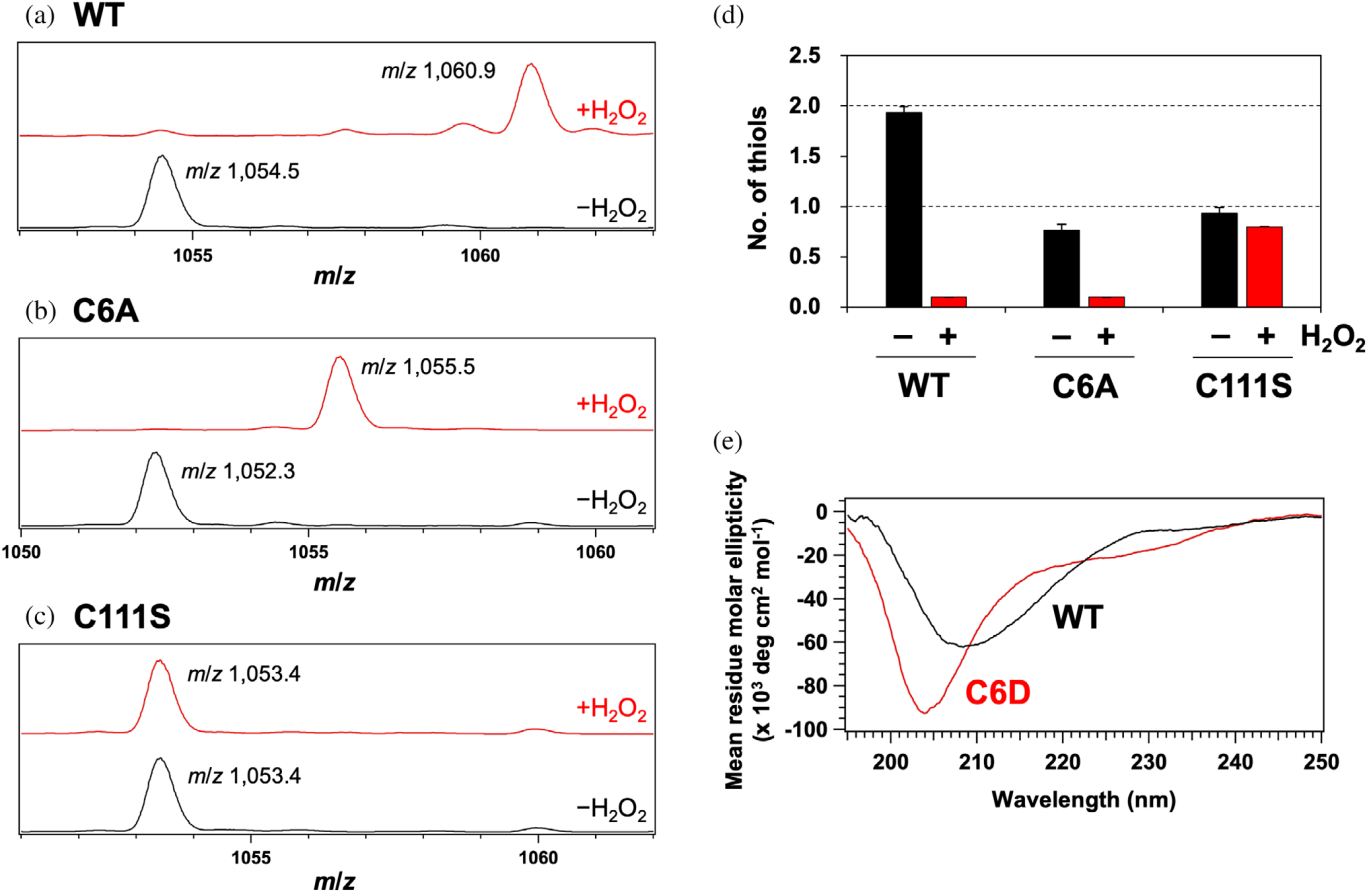
Distinct reactivities of Cys6 and Cys111 in apo‐SOD1 for oxidation with H_2_O_2_ (a–c) Mass spectra of (a) apo‐SOD1(WT), (b) apo‐SOD1(C6A), and (c) apo‐SOD1(C111S) were acquired under denaturing conditions with 50% acetonitrile and 0.1% formic acid. Peaks corresponding to the 15+ charge state are displayed. Black and red spectra represent SOD1 before and after H_2_O_2_ treatment, respectively. Full mass spectra are provided in Figure S1. (d) The number of thiol groups per SOD1 monomer for apo‐SOD1(WT, C6A, C111S) treated with or without H_2_O_2_ was measured using the DTNB assay. Data are presented as the mean ± standard deviation from three independent experiments. (e) CD spectra of (black) apo‐SOD1(WT) and (red) apo‐SOD1(C6D) were recorded in 5 mM MOPS/100 mM NaCl (pH 7.0). For this experiment, SOD1 proteins with reduced disulfide bonds were used, and the N‐terminal 6× His tag remained uncleaved.

To further characterize oxidation in SOD1 upon exposure to H_2_O_2_, mass spectrometry analysis was conducted on apo‐SOD1 before and after the oxidation (Figure S2). As shown in Figure 3a (black line), the peak at *m*/*z* 1054.5 corresponds to the 15+ charge state of apo‐SOD1 with the conserved disulfide bond, yielding an observed mass of 15,802 Da, which matches the calculated mass (15,802 Da). Following the oxidation with H_2_O_2_, the 15+ charge state peak shifted to *m*/*z* 1060.9, corresponding to an observed mass of 15,898 Da (Figure 3a, red line). This mass increase of 96 Da suggests the incorporation of six oxygen atoms upon the oxidation.

Among amino acids, cysteine is the most susceptible to oxidation (Lo Conte & Carroll, 2013). SOD1 contains two free cysteine residues, Cys6 and Cys111 (Figure 1), with Cys111 being particularly prone to oxidation by H_2_O_2_ (Fujiwara et al., 2007). To determine whether these cysteine residues were oxidized in this study, the thiol content of apo‐SOD1 before and after the oxidation was quantified using DTNB (Ellman's reagent). In apo‐SOD1 before the oxidation, the number of thiol groups was determined to be 1.9, consistent with the presence of two free cysteine residues, Cys6 and Cys111 (Figure 3d). Following the oxidation with H_2_O_2_ for 3 h, the thiol content decreased to 0.5, and further declined to 0.1 after 24 h, indicating that both Cys6 and Cys111 were ultimately oxidized. Furthermore, previous studies have reported that a copper‐ligating His residue is oxidized to 2‐oxo‐histidine when holo‐SOD1 is treated with 5 mM H_2_O_2_ (Uchida & Kawakishi, 1994). Because 2‐oxo‐histidine can be resolved from histidine by amino acid analysis (Lewisch & Levine, 1995), we analyzed apo‐SOD1 before and after treatment with 10 mM H_2_O_2_ for 24 h. No 2‐oxo‐histidine was detected after oxidation, and the observed His‐to‐Arg ratios (1.95 and 1.96 before and after oxidation, respectively) were essentially identical to the theoretical value of 2, where Arg was used as a non‐oxidizable reference amino acid. In contrast, cysteic acid was detected at a relative abundance of 0.43 per Arg, closely matching the theoretical value of 0.5 expected when both Cys6 and Cys111 are oxidized to sulfonic acid. These results demonstrate that, under our conditions, His residues in apo‐SOD1 are not oxidized by H_2_O_2_; instead, oxidation targets Cys residues.

To investigate the roles of cysteine oxidation in the SOD1 denaturation, apo‐SOD1 carrying the C6A substitution was oxidized with H_2_O_2_. Upon oxidation, the observed mass increased from 15,769 (*m*/*z* 1052.3 of the 15+ charge state) to 15,817 (*m*/*z* 1055.5 of the 15+ charge state), corresponding to an addition of three oxygen atoms (48 Da) (Figure 3b). In addition, the number of thiol groups quantified using DTNB decreased from 0.8 to 0.1 after 24 h of oxidation (Figure 3d) with this decrease already complete within the first 3 h. Consistent with the previous study (Fujiwara et al., 2007), these results indicate that Cys111 in SOD1(C6A) was oxidized to sulfonic acid (–SO_3_H). Nonetheless, the CD spectrum of apo‐SOD1(C6A), which closely overlapped with that of apo‐SOD1, remained almost unchanged upon oxidation (Figure S3A). These findings suggest that the oxidation of Cys111 to sulfonic acid alone does not directly induce SOD1 denaturation.

Unexpectedly, the oxidation of apo‐SOD1 carrying the C111S substitution with H_2_O_2_ did not result in any detectable mass shift (15,786, *m*/*z* 1053.4 of the 15+ charged state) (Figure 3c). Similarly, the number of thiol groups quantified using DTNB remained nearly unchanged after 24 h of oxidation (0.9 before and 0.8 after; Figure 3d). These results indicate that in SOD1(C111S), all amino acid residues, including Cys6, remained unmodified following the H_2_O_2_ treatment. Furthermore, the CD spectrum of apo‐SOD1(C111S), which closely resembled that of apo‐SOD1, remained unchanged upon H_2_O_2_ exposure (Figure S3B). Collectively, these findings suggest that Cys6 oxidation requires prior oxidation of Cys111 and that Cys6 oxidation is likely a key event driving SOD1 denaturation.

### 2.3 Oxidation of Cys6 denatures SOD1

To further investigate how Cys6 oxidation impacts SOD1 structure, we generated a C6D variant, in which the aspartic acid side chain serves as a structural mimic of sulfinic acid (–SO_2_H). While this substitution would not perfectly replicate sulfonic acid (Garrido Ruiz et al., 2022), it provides a reasonable model for the increased hydrophilicity and introduction of a negative charge associated with thiol oxidation.

We initially aimed to evaluate the structure of SOD1(C6D) while maintaining the conserved disulfide bond between Cys57 and Cys146 (Figure 1). To achieve this, we expressed the protein in *Escherichia coli* SHuffle™, a strain that facilitates disulfide bond formation. However, purification proved challenging, as SOD1(C6D) was primarily obtained as disulfide‐linked oligomers (data not shown), suggesting that the C6D substitution promotes aberrant intermolecular disulfide formation, possibly due to the significant structural destabilization. This challenge prevented us from obtaining a well‐defined SOD1(C6D) sample with the conserved disulfide bond. As an alternative approach, therefore, we chose to examine the structure of SOD1(C6D) in its disulfide‐reduced form. To this end, we expressed the protein with an N‐terminal His tag in *E. coli* BL21(DE3), where the cytoplasmic environment is reducing. The His‐tagged SOD1(C6D) was then purified and treated with TCEP to ensure the absence of disulfide bonds.

To assess the structural effects of the C6D substitution, CD spectroscopy was performed using the purified SOD1(C6D) alongside His‐tagged wild‐type SOD1 in the apo and disulfide‐reduced state. As shown in Figure 3e, the wild‐type SOD1 protein exhibited a CD spectrum comparable to that of non‐tagged apo‐SOD1 with the conserved disulfide bond; in contrast, SOD1(C6D) displayed a spectrum similar to that of H_2_O_2_‐oxidized SOD1, suggesting that the oxidation of Cys6 causes structural destabilization of SOD1. Cys6 is a key component of the hydrophobic core in the β‐barrel structure of SOD1, and the oxidation of Cys6 is thus expected to disrupt this core, leading to structural destabilization and ultimately denaturation.

### 2.4 Molecular dynamics (MD) simulation predicts weakened interaction between subunits in SOD1 upon oxidation of Cys111

To assess the impact of Cys111 oxidation on the structural properties of SOD1, MD simulations were performed. The appropriate protonation state of Cys111 for the simulations was first expected based upon the p*K*_a_ values of the thiol/thiolate and sulfonic acid/sulfonate forms. Using a program pKAI (Reis et al., 2022), the p*K*_a_ of the thiol/thiolate group at Cys111 was calculated to be 9.8, indicating that Cys111 predominantly exists as a thiol under physiological conditions. In contrast, the p*K*_a_ of the sulfonic acid group is generally low, ~1.3 in cysteic acid, suggesting that oxidized Cys111 primarily exists as a sulfonate at physiological pH. Apo‐SOD1 was thus modeled with Cys111 in its thiol form, while oxidized SOD1 was modeled with Cys111 in its sulfonate form.

Simulations were then conducted at 310 K for 1.0 μs with three independent replicates. During the simulations, root‐mean‐square deviation (RMSD) analysis indicated that the overall structure of each SOD1 subunit, including the region surrounding Cys111, remained largely unchanged, except for the electrostatic loop (Glu121–Ser142, Figure 1), which is intrinsically flexible (Figure S4A,B). Furthermore, oxidation at Cys111 did not significantly influence the temporal changes of the loop structure. Root‐mean‐square fluctuation (RMSF) analysis also demonstrated that the structural flexibility of SOD1 was not substantially affected by the oxidation of Cys111 (Figure S4C). These findings thus suggest that sulfonylation at Cys111 does not induce major conformational changes in the folded structure of SOD1.

**FIGURE 4:**
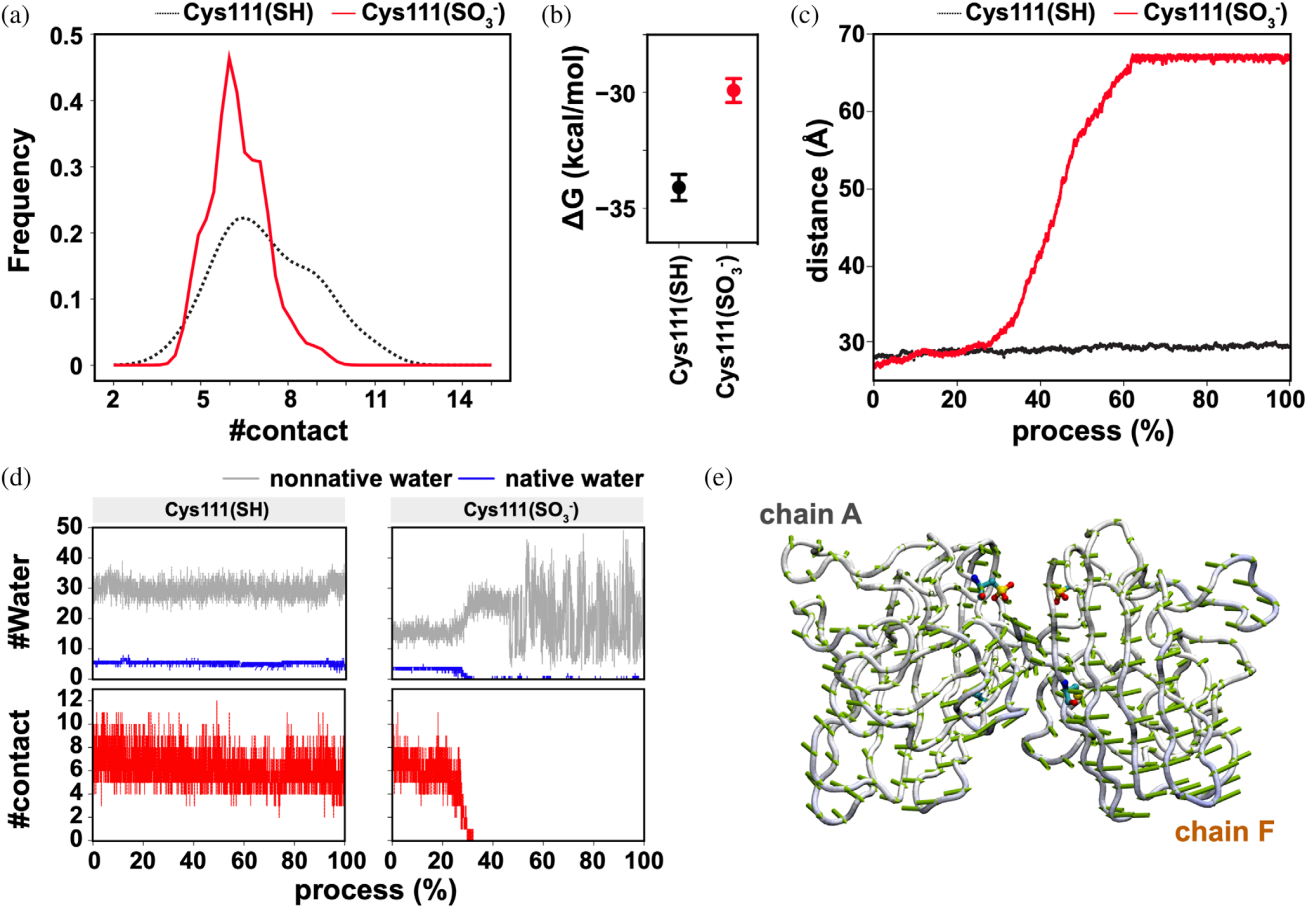
Classical MD simulation and dissociation analysis of dimeric SOD1 (a) The number of atomic contacts (#contact) and (b) the binding free energy (Δ*G*) between chain A and F were evaluated using the MM/GBSA method. (c) The distance profiles during the dissociation process are shown for SOD1 with the thiol (Cys111(SH)) (black line) and the sulfonate (Cys111(SO_3_^−^)) (red line) at Cys111, highlighting the differences in separation dynamics. (d) The number of water molecules (#Water) between chain A and F was monitored throughout the simulation, distinguishing between native (blue line) and non‐native water (gray line), alongside changes in atomic contacts (#contact). (e) The porcupine plot (green arrows) illustrates the direction of dissociation between chain A and F, revealing the principal motions contributing to dimer separation.

Given that the side chain of Cys111 resides at the dimer interface of SOD1 (Figure 1), we hypothesized that its oxidation might disrupt subunit interactions. Consistent with this hypothesis, the number of atomic contacts between subunits was reduced during the simulation when Cys111 was in the sulfonate state compared with the thiol state (Figure 4a). The absolute value of the free energy of subunit interaction was also decreased upon oxidation of Cys111 from thiol to sulfonate (Figure 4b). These findings thus predict that Cys111 oxidation to sulfonate facilitates SOD1 monomerization.

To further assess whether the interaction between SOD1 subunits is disrupted by the sulfonylation of Cys111, dissociation simulations were performed. Analysis of the center‐of‐mass distance between the subunits revealed that SOD1 with Cys111 in the sulfonate state underwent a more pronounced dissociation process compared with the thiol state during the simulations (Figure 4c). Simultaneously, atomic contacts at the dimer interface were significantly reduced in the sulfonate state (Figure 4d, bottom). Furthermore, PCA analysis demonstrated that the two subunits in the sulfonate state gradually separated through a twisting motion, eventually dissociating into monomers (Figure 4e). In the PCA scatter plot, the separation of the subunits is reflected by the significant movement along the principal components, showing large‐scale conformational changes as the protein shifts toward a dissociated state (Figure S5A). Notably, the number of water molecules at the dimer interface increased during the simulations when Cys111 was in the sulfonate state (Figure 4d, top). The conversion from a hydrophobic thiol to a highly hydrophilic sulfonate at Cys111 likely promotes the influx of water molecules at the dimer interface, with non‐native water molecules being introduced and disrupting the native hydration shell (Figure 4d, top). This water‐mediated disruption weakens the hydrophobic interactions crucial for maintaining the dimer interface, ultimately leading to complete subunit separation (Movie S1). Importantly, such water‐induced destabilization was not observed in wild‐type SOD1 with Cys111 in the thiol state (Figure 4c,d) or even when Cys111 was replaced with the hydrophilic amino acid Ser (Figure S5B,C), underscoring the unique impact of sulfonylation on the stability of the SOD1 dimer.

### 2.5 Oxidation of Cys111 promotes monomerization of SOD1

**FIGURE 5:**
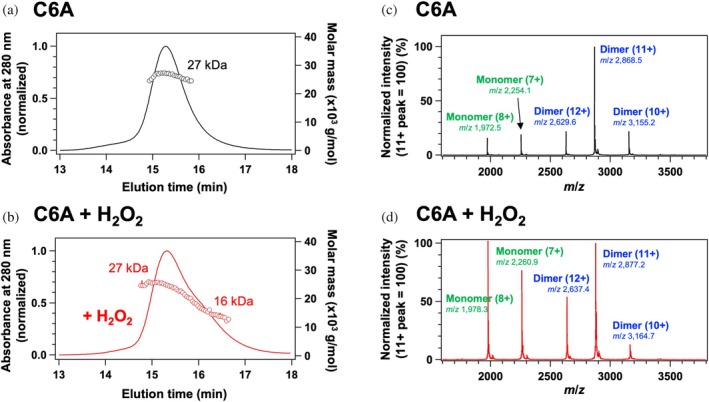
Oxidation of Cys111 facilitates the monomerization of apo‐SOD1 (a, b) Gel filtration chromatograms of 20 μM apo‐SOD1(C6A) in the MN buffer (a) before and (b) after treatment with 10 mM H_2_O_2_, monitored at 280 nm and normalized for comparison (solid lines, left axis), along with molecular masses estimated by MALS (circles, right axis). (c, d) Mass spectra of apo‐SOD1(C6A) under native conditions (c) before and (d) after treatment with H_2_O_2_. The intensity of each spectrum was normalized to set the peak corresponding to the 11+ charge state of the dimer to 100%.

To experimentally validate the monomerization of SOD1 upon Cys111 oxidation, as predicted by MD simulations, we prepared SOD1 with sulfonylated Cys111 by oxidizing apo‐SOD1(C6A) with H_2_O_2_. SEC‐MALS analysis revealed that non‐oxidized SOD1(C6A) eluted as a single peak with a molecular mass of 27 kDa, confirming its homodimeric state (Figure 5a). In contrast, oxidized SOD1(C6A) displayed a similar elution profile but with an additional shoulder at a later elution time (Figure 5b). MALS analysis indicated a molecular mass of 16 kDa for this fraction, suggesting that the sulfonylation of Cys111 facilitates SOD1 monomerization.

We further investigated the increased monomerization tendency of SOD1 upon Cys111 sulfonylation using native mass spectrometry. Based on peak assignments established in our previous study (Tajiri et al., 2022), apo‐SOD1(C6A) under native conditions predominantly exhibited mass peaks corresponding to the homodimer, with significantly higher intensity than those of the monomeric state, consistent with its dimeric nature (Figure 5c). In contrast, when SOD1(C6A) was oxidized with H_2_O_2_, the relative intensity of the monomeric peaks increased markedly compared with the dimeric peaks (Figure 5d). These results further support the notion that oxidation at Cys111 promotes SOD1 monomerization.

### 2.6 Monomerization enhances the susceptibility of Cys6 to H_2_O_2_‐mediated oxidation

**FIGURE 6:**
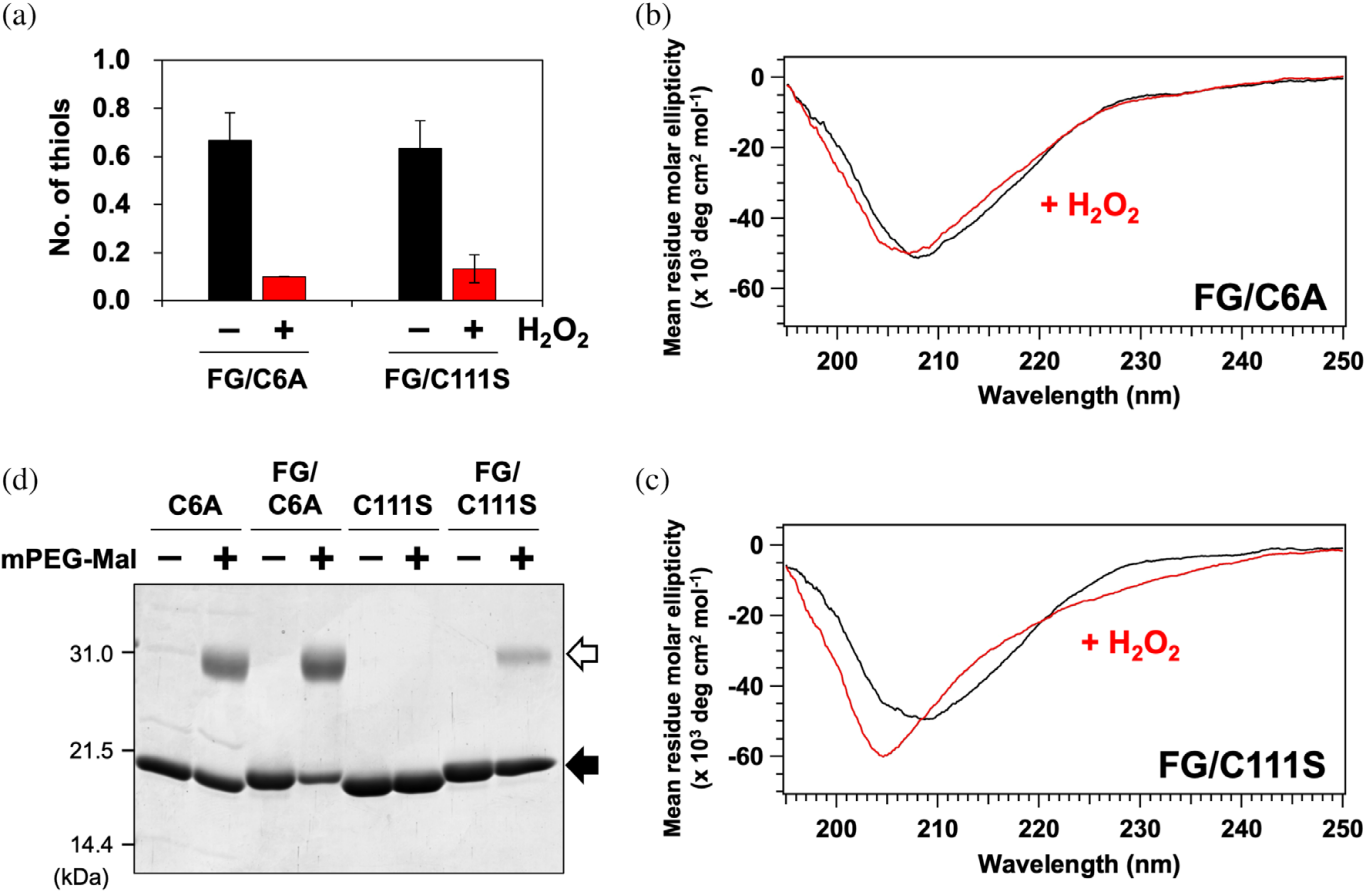
Cys6 in apo‐SOD1 is susceptible to oxidation in the monomeric state (a) The number of thiol groups per SOD1 monomer for apo‐SOD1(FG/C6A, FG/C111S) treated with or without H_2_O_2_ was measured using the DTNB assay. Data are presented as the mean ± standard deviation from three independent experiments. (b, c) CD spectra of (b) 20 μM apo‐SOD1(FG/C6A) and (c) 20 μM apo‐SOD1(FG/C111S) in 5 mM MOPS/100 mM NaCl (pH 7.0) are shown (black) before and (red) after treatment with 10 mM H_2_O_2_. (d) Apo‐SOD1 variants before (−) and after (+) modification with mPEG‐MAL were analyzed by SDS‐PAGE. The black arrow indicates unmodified SOD1 bands, while the white arrow indicates mPEG‐MAL‐modified SOD1 bands.

Given that Cys111 oxidation promotes monomerization, the resulting monomeric state may facilitate the oxidation of Cys6 by H_2_O_2_. To test this hypothesis, we examined the oxidation of Cys6 in a monomeric SOD1 variant carrying the F50E/G51E (FG) substitutions (Bertini et al., 1994), into which an additional substitution (C6A or C111S) was introduced (FG/C6A, FG/C111S). Analysis by SEC‐MALS confirmed that both apo‐SOD1(FG/C6A) and apo‐SOD1(FG/C111S) existed in a monomeric state (Figure S6). Quantification of thiol groups by the DTNB assay indicated approximately 0.7 thiols per protein molecule for both variants (Figure 6a). As expected from observations in the dimeric state, Cys111 in monomeric apo‐SOD1(FG/C6A) was readily oxidized by H_2_O_2_, as evidenced by the loss of thiol groups (Figure 6a). Notably, apo‐SOD1(FG/C111S), lacking Cys111, also exhibited a loss of thiol groups upon oxidation (Figure 6a), clearly demonstrating that oxidation of Cys6 in the monomeric state occurs independently of prior Cys111 oxidation.

Furthermore, CD spectroscopy revealed that the spectra of those monomeric variants closely resembled that of wild‐type SOD1, characterized by a negative trough at 210 nm, albeit with slightly reduced intensity (Figure 6b,c). Oxidation of apo‐SOD1(FG/C6A) with H_2_O_2_ did not significantly alter its CD spectrum, indicating that oxidation of Cys111 alone does not induce structural denaturation of SOD1 even in the monomeric state (Figure 6b). In contrast, oxidation of apo‐SOD1(FG/C111S) resulted in notable spectral changes, including a shift in the negative trough from 210 to 205 nm and a less steep slope around 225 nm—similar to those observed upon oxidation of apo‐SOD1 (Figure 6c). Taken together, these findings demonstrate that the monomerization triggered by oxidation of Cys111 facilitates subsequent oxidation of Cys6 by H_2_O_2_, ultimately leading to loss of structural integrity in SOD1.

To investigate why the monomerization facilitates Cys6 oxidation, we performed thiol modification using mPEG‐MAL to assess the accessibility of cysteine side chains. In both SOD1(C6A) and the monomeric variant SOD1(FG/C6A), a band with reduced electrophoretic mobility, corresponding to SOD1 modified with mPEG‐MAL, was clearly observed (Figure 6d), indicating that Cys111 is readily accessible in both dimeric and monomeric states. In contrast, SOD1(C111S) was not modified by mPEG‐MAL, whereas its monomeric variant, SOD1(FG/C111S), exhibited a distinct band corresponding to mPEG‐MAL modification (Figure 6d). These findings suggest that monomerization increases the solvent exposure of Cys6, making it more accessible to H_2_O_2_ and thereby more susceptible to oxidation.

### 2.7 Zinc binding protects SOD1 from oxidative denaturation

Our experiments thus far have focused on apo‐SOD1 to understand its oxidative denaturation. Given that SOD1 normally binds a zinc ion under physiological conditions, it is crucial to examine the influence of Zn^2+^ binding on the oxidation of Cys6 in SOD1. Native SOD1 also binds a copper ion, and a copper‐bound, Zn^2+^‐deficient form of SOD1 has been proposed to exert toxicity by promoting superoxide production and generating peroxynitrite (Estevez et al., 1999). As noted in the Introduction, however, copper ions can induce nonspecific oxidative degradation of SOD1 upon reaction with H_2_O_2_ (Ookawara et al., 1992). Furthermore, copper deficiency in neuronal SOD1 is plausible under certain pathological conditions (Abdeen et al., 2025; Hilton et al., 2024; Trist et al., 2022). Therefore, we next investigated how Zn^2+^ binding affects the oxidation of Cys6, aiming to clarify the conditions under which oxidative denaturation of SOD1 can take place.

**FIGURE 7:**
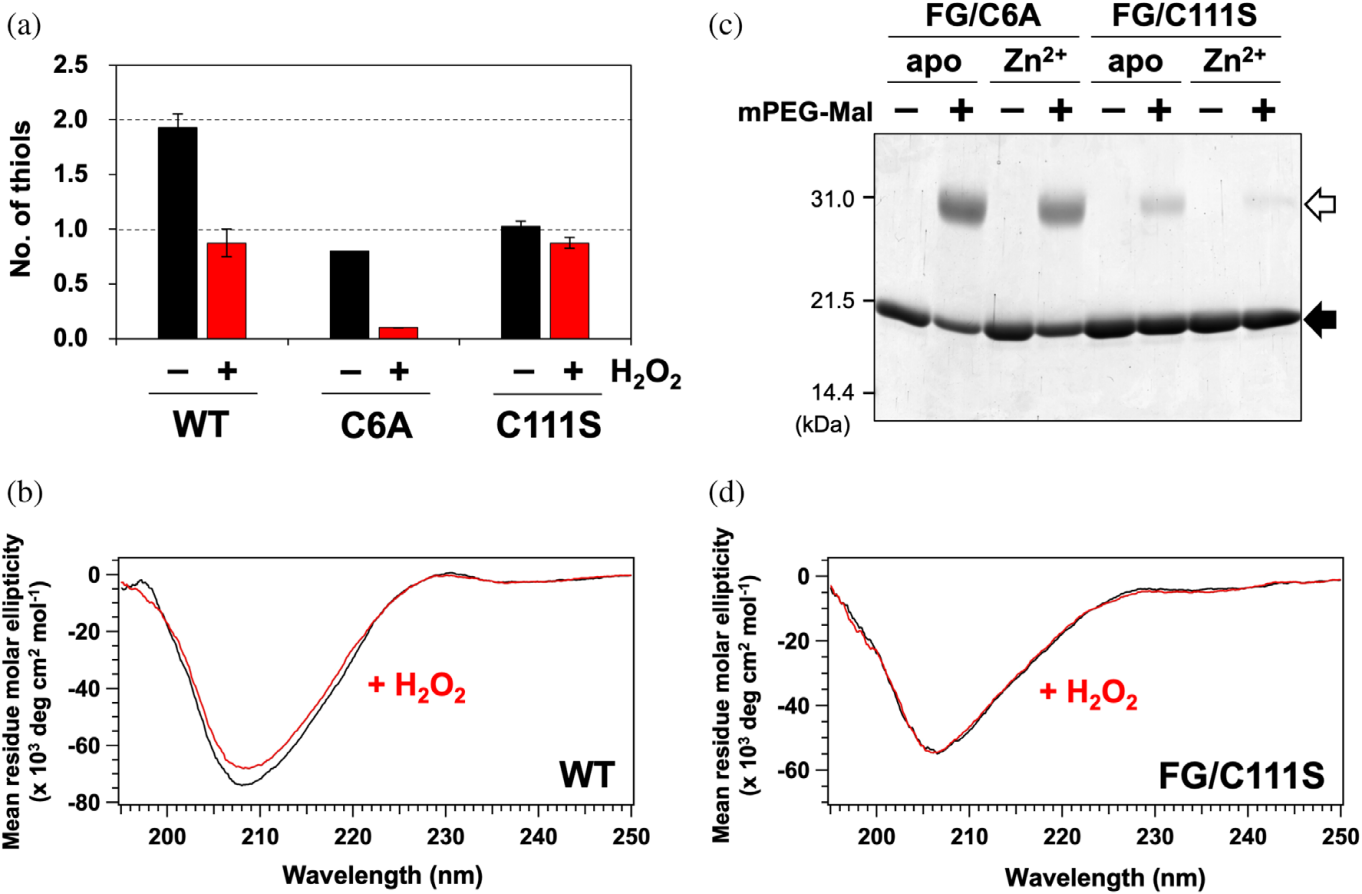
Binding of Zn^2+^ protects SOD1 from oxidation at Cys6. (a) The number of thiol groups per SOD1 monomer for SOD1(WT, C6A, C111S) in the presence of Zn^2+^, treated with or without H_2_O_2_, was measured using the DTNB assay. Data are presented as the mean ± standard deviation from three independent experiments. (b, d) CD spectra of (b) 20 μM SOD1(WT) and (d) 20 μM SOD1(FG/C111S) in the presence of Zn^2+^ in 5 mM MOPS/100 mM NaCl (pH 7.0). Spectra are shown for samples (black) before and (red) after treatment with 10 mM H_2_O_2_. (c) SOD1 variants with and without Zn^2+^, before (−) and after (+) modification with mPEG‐MAL, were analyzed by SDS‐PAGE. The black arrow indicates unmodified SOD1 bands, while the white arrow indicates mPEG‐MAL‐modified SOD1 bands.

In the presence of Zn^2+^, oxidation of SOD1 by H_2_O_2_ resulted in a decrease in thiol groups from 1.9 to 0.9, as determined by the DTNB assay (Figure 7a). This result contrasts sharply with the complete loss of thiol groups observed upon oxidation of apo‐SOD1 (Figure 3d), suggesting a protective effect by Zn^2+^ binding. To clarify which cysteine residue remains unoxidized, similar experiments were performed using Zn^2+^‐bound SOD1 variants with a substitution at either Cys6 or Cys111. Upon oxidation, Zn^2+^‐bound SOD1(C6A) exhibited a marked reduction in thiol groups (from 0.8 to 0.1), whereas Zn^2+^‐bound SOD1(C111S) showed minimal change (from 1.0 to 0.9) (Figure 7a). These results demonstrate that Zn^2+^‐bound SOD1 selectively allows oxidation of Cys111 while effectively protecting Cys6 from oxidation.

This selective oxidation of Cys111, while Cys6 remains unaffected, differs from the oxidation sequence observed in apo‐SOD1, where Cys111 oxidation facilitated subsequent oxidation of Cys6, ultimately leading to denaturation. Consistently, the CD spectrum of Zn^2+^‐bound wild‐type SOD1 showed no significant changes before and after oxidation with H_2_O_2_ (Figure 7b). Furthermore, Zn^2+^‐bound SOD1(WT, C6A) remained dimeric even after oxidation with H_2_O_2_ (Figure S7).

To test whether the suppression of denaturation is attributed to the stabilization of the dimeric state upon Zn^2+^ binding, we examined the oxidation of Cys6 in monomeric Zn^2+^‐bound SOD1(FG/C111S). Notably, the number of thiol groups in Zn^2+^‐bound SOD1(FG/C111S) remained largely unchanged upon oxidation with H_2_O_2_ (from 0.63 ± 0.10 to 0.53 ± 0.13), despite being slightly lower than that expected in the unoxidized protein. Thiol modification with mPEG‐MAL revealed that Zn^2+^ binding had distinct effects on solvent accessibility: while SOD1(FG/C6A) remained efficiently modified, modification of SOD1(FG/C111S) was significantly retarded in the presence of Zn^2+^ (Figure 7c). This suggests that Zn^2+^ binding reduces the solvent exposure of Cys6 even in the monomeric state. Furthermore, the CD spectrum of Zn^2+^‐bound SOD1(FG/C111S) showed no significant alterations before and after the oxidation (Figure 7d). These results indicate that Zn^2+^ binding prevents Cys6 oxidation and subsequent denaturation, even in the monomeric state of SOD1.

Based upon those findings, we propose that the dissociation of Zn^2+^, followed by Cys111 oxidation or vice versa, promotes the formation of monomeric apo‐SOD1. This structural transition is considered to result in the increased fluctuations around Cys6, making it more susceptible to oxidation. The subsequent oxidation of Cys6 ultimately disrupts structural integrity, leading to the denaturation of SOD1.

### 2.8 Effects of pathogenic substitutions on the oxidative denaturation of SOD1

**FIGURE 8:**
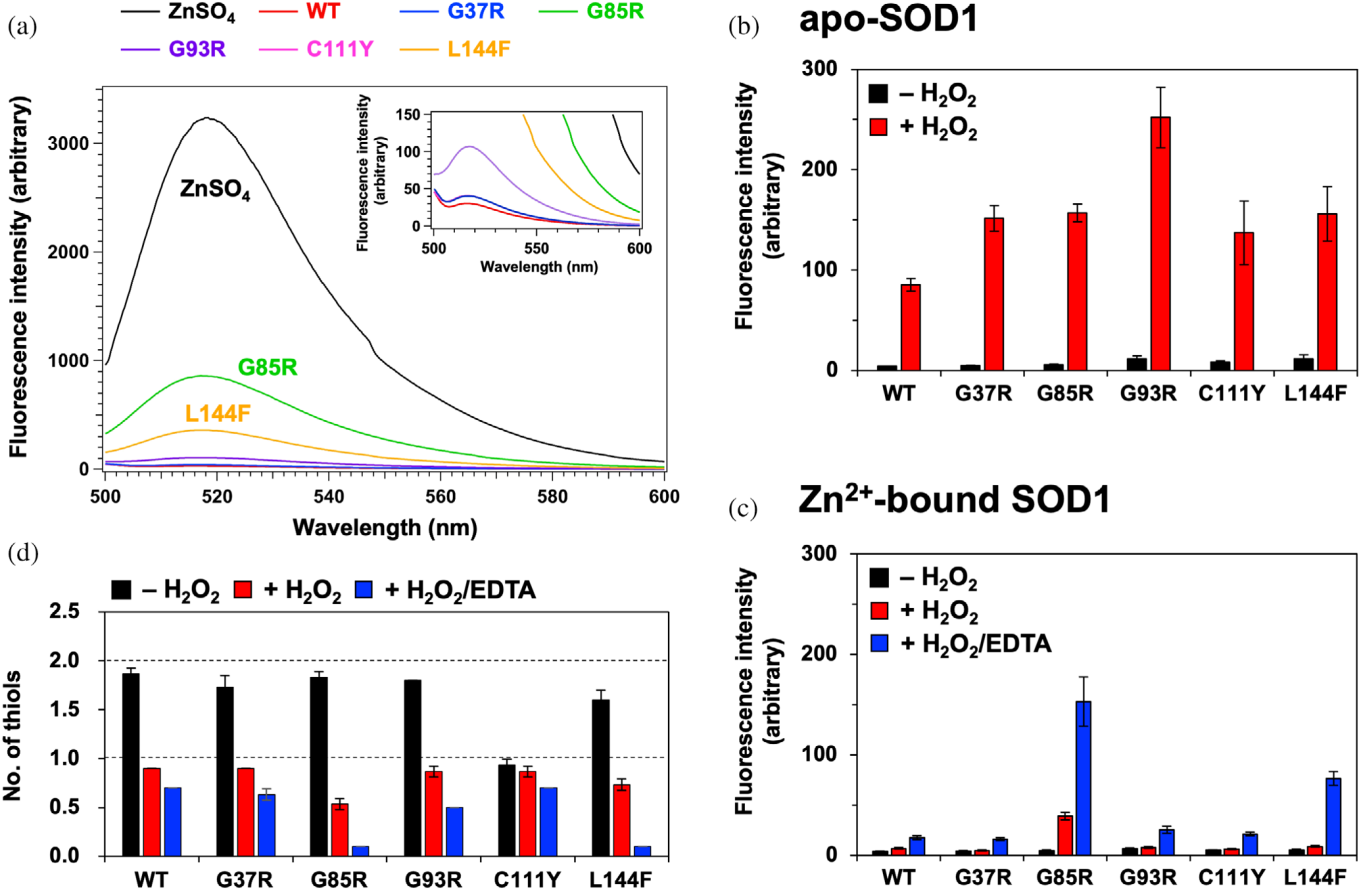
Oxidative denaturation preferentially occurs in SOD1 variants with pathogenic mutations that decrease Zn^2+^ affinity (a) Fluorescence spectra of 10 μM ZnAF‐2 with 10 μM SOD1 variants pre‐incubated with an equimolar amount of ZnSO_4_. The spectrum of 10 μM ZnAF‐2 with 10 μM ZnSO_4_ in the absence of SOD1 is shown as “ZnSO_4_” and serves as a control. Magnified spectra are also displayed in the inset. (b, c) Fluorescence intensities of SYPRO™ Orange are plotted for (b) apo‐SOD1 and (c) Zn^2+^‐bound SOD1 variants (black) before and (red) after treatment with H_2_O_2_. For the experiments using Zn^2+^‐bound SOD1 variants, fluorescence intensities of samples oxidized with H_2_O_2_ in the presence of EDTA are also plotted and displayed as blue bars. (d) The number of thiol groups per SOD1 monomer for the indicated SOD1 variants in the presence of Zn^2+^ was measured using the DTNB assay under three conditions: (black) untreated, (red) treated with H_2_O_2_, and (blue) treated with H_2_O_2_ in the presence of EDTA. Data presented in (b–d) represent the mean ± standard deviation from three independent experiments.

The Zn^2+^ affinity of SOD1 is thought to be compromised by certain ALS‐associated mutations, potentially predisposing SOD1 to oxidative denaturation by promoting the formation of an apo state. The Zn^2+^ affinity of SOD1 is, however, extremely high (*K*_d_ ~ 4.8 × 10^−11^) (Leal et al., 2015), making it difficult to measure accurately, and even some of the ALS‐associated SOD1 mutants appear to retain tightly bound Zn^2+^ (Hayward et al., 2002). We thus first examined the impact of ALS‐associated mutations on Zn^2+^ binding in SOD1 by using ZnAF‐2, a fluorescent probe with strong and specific affinity for Zn^2+^ (*K*_d_ = 2.7 nM) (Hirano et al., 2000). A Zn^2+^‐bound form of SOD1 was prepared by incubating apo‐SOD1 with an equimolar concentration of ZnSO_4_, followed by mixing with an equimolar amount of ZnAF‐2. As shown in Figure 8a, ZnAF‐2 exhibited fluorescence upon binding Zn^2+^ (indicated as “ZnSO_4_”); however, wild‐type SOD1 and ALS‐associated mutants G37R, G93R, and C111Y showed almost no fluorescence even after 48 h of incubation with ZnAF‐2, suggesting that Zn^2+^ remained tightly bound. In contrast, the ALS‐associated mutants G85R and L144F exhibited significantly higher fluorescence, which remained unchanged over 48 h, although still lower than the fluorescence observed in Zn^2+^ solution alone with ZnAF‐2. This suggests that while these variants can still bind Zn^2+^, their Zn^2+^ affinity is significantly reduced.

All ALS‐associated SOD1 mutants examined in this study, as well as wild‐type SOD1, exhibited a significant increase in SYPRO™ Orange fluorescence in their apo form upon oxidation with H_2_O_2_, indicating oxidative denaturation (Figure 8b). In contrast, when SOD1 variants were incubated with H_2_O_2_ in the presence of Zn^2+^, SYPRO™ Orange fluorescence remained negligible, except for the G85R mutant (Figure 8c). This aligns with our findings that Zn^2+^ binding protects SOD1 from oxidative denaturation and that the G85R mutation significantly reduces Zn^2+^ affinity (Figure 8a). Furthermore, when oxidation with H_2_O_2_ was conducted in the presence of the divalent metal chelator EDTA, both wild‐type and ALS‐associated mutant SOD1s displayed increased SYPRO™ Orange fluorescence (Figure 8c). Notably, the G85R and L144F mutants, which exhibited significantly reduced Zn^2+^ affinity, showed the highest fluorescence intensity among all variants (Figure 8c). Importantly, the fluorescence increase was more pronounced in the G85R mutant than in L144F, consistent with the greater impairment of Zn^2+^ coordination in G85R compared with L144F (Figure 8a). These results suggest that among ALS‐associated mutations, those that weaken Zn^2+^ binding specifically promote oxidative denaturation of SOD1, with the severity of denaturation correlating with the extent of Zn^2+^ binding impairment.

Regarding cysteine oxidation, the DTNB assay quantified the number of thiol groups in Zn^2+^‐bound SOD1 proteins as close to 2 before oxidation, except for C111Y, which had a value near 1 (Figure 8d, black). Upon oxidation with H_2_O_2_, the number of thiol groups decreased to approximately 1 in most variants, consistent with preferential oxidation of Cys111 (Figure 8d, red); in G85R, however, the number was significantly lower, approaching 0.5, suggesting that a substantial fraction of Cys6 was also oxidized despite the presence of Zn^2+^, which correlates with the observed oxidative denaturation (Figure 8c, red). Furthermore, when oxidation was conducted in the presence of EDTA, the number of thiol groups in Zn^2+^‐bound SOD1 further decreased, with G85R and L144F showing values close to zero, indicating Cys6 oxidation (Figure 8d, blue). These results are consistent with our findings that the G85R and L144F mutations promote oxidative denaturation of SOD1 by reducing its Zn^2+^ affinity, thereby making Cys6 more susceptible to oxidation. Taken together, these findings suggest that a subset of pathogenic mutations weakens the Zn^2+^ binding affinity of SOD1, enhances Cys6 susceptibility to oxidation, and thereby increases oxidative denaturation.

## 3 DISCUSSION

We demonstrated that SOD1 denaturation is triggered by the oxidation of Cys111 in conjunction with the dissociation of Zn^2+^. Apo‐SOD1 with oxidized Cys111 tends to dissociate into monomers, which in turn facilitates the oxidation of Cys6 and ultimately leads to protein denaturation. These findings suggest that SOD1 may become more susceptible to denaturation under conditions of elevated oxidative stress combined with Zn^2+^ deficiency in the cellular environment and/or reduced Zn^2+^ affinity due to structural alterations such as disease‐associated mutations.

The oxidative modification of Cys111 in SOD1, including glutathionylation (Wilcox et al., 2009), cysteinylation (Auclair et al., 2013), and sulfonylation/sulfenylation (Tokuda et al., 2019; Xu et al., 2018), has often been detected in human samples. While the pathogenic roles of those modifications remain unclear, glutathionylation at Cys111 is known to promote monomerization, possibly by disrupting dimer interface contacts to accommodate the glutathione moiety (Redler et al., 2011). In transgenic mice expressing ALS‐linked mutant SOD1 (H46R, G93A), sulfonylation or sulfinylation at Cys111 has been observed (Chen et al., 2012; Fujiwara et al., 2007; Nagano et al., 2015). Notably, mice expressing human SOD1 (H46R/C111S) showed significantly delayed disease onset compared with those expressing human SOD1 (H46R) (Nagano et al., 2015). In cultured cell models, moreover, the C111S mutation ameliorated cytotoxicity and aggregation of ALS‐mutant SOD1 (Cozzolino et al., 2008). These previous studies suggest that oxidative modification of Cys111 may enhance the pathogenicity of ALS‐associated mutant SOD1 proteins.

Despite the potential contribution of Cys111 oxidation to ALS pathology, it is important to note that ALS‐like symptoms still develop in model mice expressing ALS‐mutant SOD1 even when the thiol group at position 111 is absent. For example, mice expressing human SOD1(H46R/C111S) or murine SOD1, which naturally has a serine at position 111, with the G86R mutation (corresponding to the ALS‐causing G85R mutation in human SOD1), were found to develop ALS‐like symptoms (Nagano et al., 2015; Ripps et al., 1995). Furthermore, the C111Y mutation is known to cause familial ALS (Eisen et al., 2008; Suzuki et al., 2011). Although the replacement of Cys111 with Ser or Tyr may partially mimic the structural and chemical changes associated with Cys111 oxidation, these previous findings indicate that oxidation of Cys111 is not an absolute requirement for SOD1 to acquire pathogenicity. This observation aligns with our proposed mechanism, which suggests that Cys111 oxidation alone is insufficient to induce SOD1 denaturation. Instead, oxidative denaturation further requires both the absence of Zn^2+^ and subsequent oxidation of Cys6.

**FIGURE 9:**
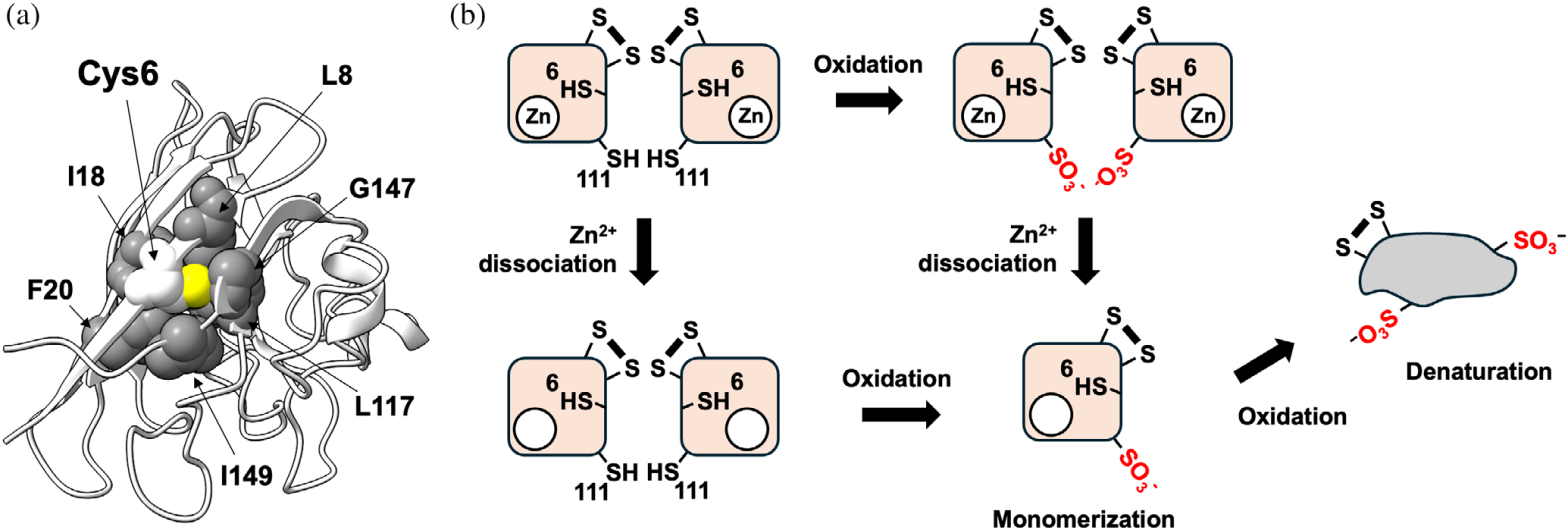
A proposed mechanism of SOD1 denaturation triggered by Cys111 oxidation (a) The side chain of Cys6, with its sulfur atom highlighted in yellow, is embedded within a hydrophobic core formed by surrounding residues shown as gray spheres. (b) Schematic illustration of the proposed pathway by which wild‐type SOD1 denatures under oxidative conditions. Oxidation at Cys111, in conjunction with the dissociation of bound metal ions, promotes subsequent oxidation of Cys6, ultimately leading to SOD1 denaturation.

Cys6 forms part of a hydrophobic core within the β‐barrel structure composed of eight β‐strands arranged in an immunoglobulin‐like fold. The side chain of Cys6 is closely packed with those of Leu8, Ile18, Phe20, Leu117, Gly147, and Ile149 (Figure 9a). Notably, we previously reported that canine SOD1, which naturally possesses Met at position 117 instead of Leu, has a smaller side chain that creates a cavity in the hydrophobic core (Hashimoto et al., 2023). This structural difference results in reduced stability compared with human SOD1, where the core is tightly packed without such a cavity. Indeed, replacing Leu117 with Met in human SOD1 decreased its thermal stability due to the formation of a cavity in the hydrophobic core (Hashimoto et al., 2023). Furthermore, several familial ALS‐associated mutations have been identified at residues forming this hydrophobic core (e.g., C6G/S/F/W, L8Q/V, L117V, G147C/D/R) (https://alsod.ac.uk). These mutations are thought to destabilize SOD1 by introducing structural perturbations that either create cavities through reduced side chain size, disrupt packing with significantly enlarged side chains, or alter the core's chemical properties from hydrophobic to hydrophilic or charged. Given those previous observations, our current study suggests that the sulfonylation at Cys6, which introduces a negative charge into the hydrophobic core, may induce destabilizing effects analogous to those caused by disease‐associated mutations. Such modifications compromise the integrity of the hydrophobic core, thereby promoting structural instability and potentially contributing to pathogenicity.

Previously, we reported that in apo‐SOD1, Cys6 is buried within the structural interior of a folded conformation at physiological temperatures, while the pathological mutation G37R markedly increases its exposure (Anzai et al., 2017). Similarly, in canine SOD1 carrying the E40K mutation, which causes degenerative myelopathy, the reactivity of Cys7 (corresponding to Cys6 in human SOD1) is increased at physiological temperatures (Shino et al., 2024). Notably, canine SOD1 naturally possesses Ser at position 111 instead of Cys, allowing the evaluation of Cys7 reactivity independently of Cys111. Furthermore, in cultured cells, Cys6 appears to play a more critical role in the aggregation propensity of human SOD1 than Cys111 (Prudencio et al., 2012). These findings suggest that, even in the absence of Cys111 oxidation, Cys6 may become susceptible to oxidation in certain pathogenic SOD1 variants. Thus, the increased reactivity of Cys6, whether due to pathogenic mutations or monomerization facilitated by Cys111 oxidation, is likely a key factor in the misfolding and aggregation processes under pathological conditions.

In conclusion, our study elucidates a mechanism of SOD1 denaturation under oxidative conditions, where Cys111 is preferentially oxidized to sulfonic acid (Figure 9b). This modification promotes monomerization in the apo state, increasing the solvent accessibility of Cys6 and thereby facilitating its oxidation, which ultimately disrupts the structural integrity of SOD1 (Figure 9b). While Cys111 is well recognized as the primary site for oxidative modification in SOD1, with its oxidation potentially leading to pathological conformations, our findings suggest that the reactivity of Cys6, rather than Cys111, plays a critical role in SOD1 misfolding. Targeting the reactivity and structural stability of Cys6 may thus provide a promising strategy for developing therapeutic approaches to prevent SOD1 misfolding and its associated pathologies.

## 4 MATERIALS AND METHODS

Electrophoresis, hydrophobicity evaluation of SOD1 based on SYPRO™ Orange fluorescence, circular dichroism spectroscopy, size exclusion chromatography with multi‐angle light scattering (SEC‐MALS), mass spectrometry, quantification of thiols by 5,5′‐dithiobis(2‐nitrobenzoic acid) (DTNB), comparative analysis on Zn^2+^‐affinity of SOD1 variants, cell viability assay, amino acid analysis, and molecular dynamics (MD) simulation are described in the Experimental Procedures in Data S1.

### 4.1 Preparation of recombinant SOD1 proteins

For the preparation of SOD1 proteins without any tags, the cDNA of human SOD1 was cloned between the NcoI and SalI site of a modified pET‐15b plasmid with the SalI site (Furukawa et al., 2010). For SOD1 fused with a 6× His tag at the N‐terminus, the cDNA of human SOD1 was cloned between the NdeI and SalI site of a modified pET‐15b plasmid with the SalI site, and the thrombin site between the 6× His tag and cloned SOD1 (LVPR/GSH) was replaced with the TEV protease site (ENLYFQ/G). Mutations were introduced by an inverse PCR method using PrimeSTAR Max DNA polymerase (TAKARA). All constructs used in this study were confirmed by DNA sequencing.

Most of the SOD1 proteins used in this study were expressed in a tag‐free form in *E. coli* SHuffle™ (New England Biolabs) and purified as previously described (Tajiri et al., 2022). For the preparation of SOD1 variants harboring C6A/F50E/G51E and F50E/G51E/C111S mutations, the proteins fused with the N‐terminal 6× His tag were expressed in *E. coli* BL21(DE3) by inducing expression with 0.4 mM isopropyl β‐D‐1‐thiogalactopyranoside (IPTG) in the presence of 2 mM CuSO_4_ to facilitate the formation of the intramolecular disulfide bond. Purification followed a previously described protocol (Anzai et al., 2017), with the exception that TEV protease was used instead of thrombin. Specifically, the N‐terminal 6× His tag in the demetallated proteins was cleaved by incubation with TEV protease at 20°C for 40 h. The TEV protease, cleaved tag, and uncleaved proteins were subsequently removed by passing the samples through a cOmplete™ His‐Tag Purification Column (1 mL, Roche). The proteins contained significant amounts of copper and zinc ions and were therefore precipitated with 20% trichloroacetic acid to remove these metal ions. The resulting pellets were washed with acetone and resolubilized with 100 mM 3‐(*N*‐morpholino) propanesulfonic acid (MOPS) and 50 mM NaCl at pH 7.0 (called MN buffer in this study). For the preparation of SOD1 with C6D mutation, the protein fused with the N‐terminal 6× His tag was expressed in *E. coli* BL21(DE3) by inducing expression with 0.4 mM IPTG. Purification again followed a previously described protocol (Anzai et al., 2017), but the proteins were used without the removal of the N‐terminal tag.

All of the purified SOD1 proteins except SOD1(C6D) were prepared as the apo form with the intramolecular disulfide bond in the MN buffer, and the concentration of SOD1 was determined spectrophotometrically at 280 nm using a molar extinction coefficient of 5625 M^−1^ cm^−1^ (7115 M^−1^ cm^−1^ for SOD1(C111Y)). SOD1(C6D) was prepared as the apo form without the disulfide bond in the MN buffer, and its molar extinction coefficient at 280 nm was 5500 M^−1^ cm^−1^. The absence of copper and zinc ions (<1%) in the apo protein samples was confirmed by graphite furnace atomic absorption spectroscopy (AA‐7000, Shimadzu). To prepare the zinc‐bound form of SOD1, apo‐SOD1 in the MN buffer was incubated with 1.5 molar equivalents of ZnSO_4_ at 37°C for an hour. SOD1 oxidation was carried out by incubating 20 μM protein with 10 mM H_2_O_2_ in the MN buffer at 37°C for 24 h, followed by treatment with 0.03 g/L catalase at 37°C for 10 min to remove residual H_2_O_2_.

## AUTHOR CONTRIBUTIONS

**Moeno Yoshida:** Methodology; investigation; writing – original draft. **Norifumi Muraki:** Investigation. **Michiko Tajiri:** Investigation. **Kowit Hengphasatporn:** Investigation; software. **Kaori Sue:** Investigation; resources; validation. **Takehiro Kubo:** Investigation. **Satoko Akashi:** Resources. **Yasuteru Shigeta:** Resources. **Taiho Kambe:** Resources. **Yoshiaki Furukawa:** Conceptualization; project administration; funding acquisition; writing – original draft; writing – review and editing.

## CONFLICT OF INTEREST STATEMENT

The authors declare no conflicts of interest.

## Supporting information


**Data S1.** Supporting Information.

**Movie S1.** Dimer dissociation process of SOD1 with sulfonated Cys111 based on the LB‐PaCS‐MD simulation. The two subunits of SOD1 are shown in black and orange for chains A and F, respectively. The side chain of Cys111, in which the thiol group is oxidized to a sulfonate, is displayed as space‐filling models. The oxygen atom of water molecules within 6 Å of Cys111 are shown as red spheres.

## Data Availability

The data that support the findings of this study are available from the corresponding author upon reasonable request.
