# Supplementary material for: Oxidative denaturation of Cu/Zn‐superoxide dismutase associated with neurodegenerative diseases
Source: Protein Sci. 2025 Oct 16;34(11):e70339. doi: 10.1002/pro.70339 (PMC12529874; doi:10.1002/pro.70339)
Supplement: Supplementary file 1 — Data S1. Supporting Information. [file PRO-34-e70339-s001.pdf]

Supplemental Figures

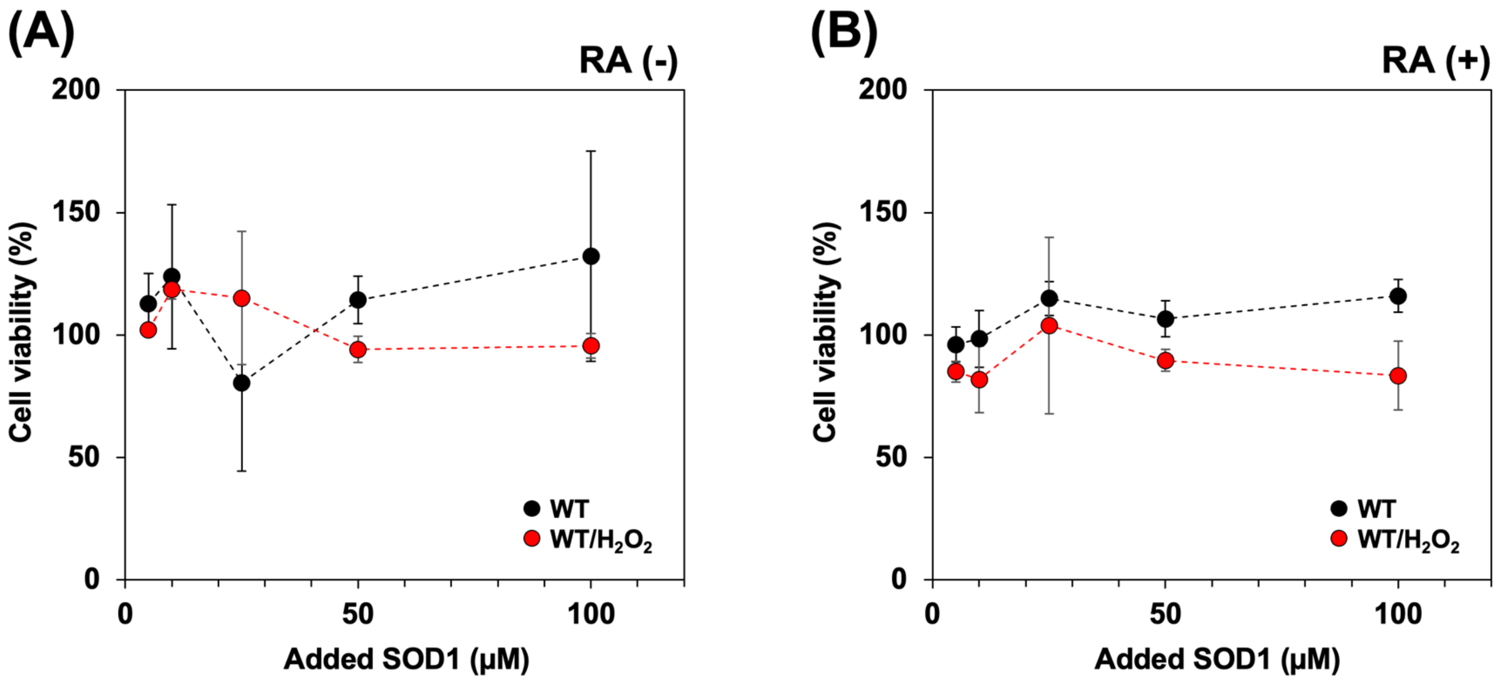

Figure S1 Oxidized apo-SOD1 did not significantly affect cell viability The viability of SH-SY5Y cells treated with the indicated concentration of (black) SOD1(WT) and (red) SOD1(WT) oxidized with H_2O_2 in the **(A)** absence and **(B)** presence of 10 µM retinoic acid (RA). Data are presented as the mean ± SD (n=3).

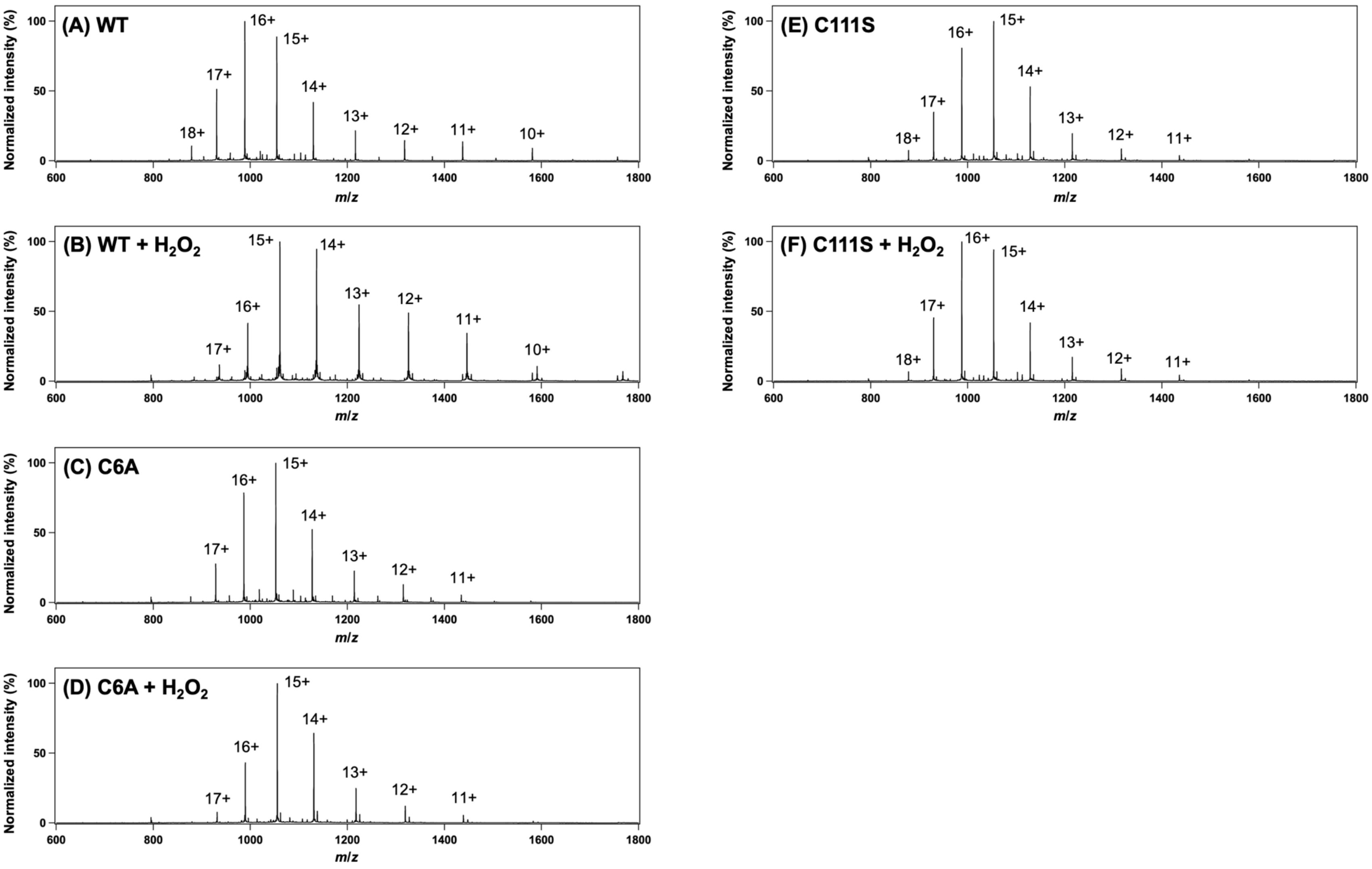

Figure S2 Characterization of SOD1 oxidation by mass spectrometry under denaturing conditions Mass spectra of apo-SOD1 variants obtained under denaturing conditions with 50% acetonitrile and 0.1% formic acid: **(A, B)** apo-SOD1(WT), **(C, D)** apo-SOD1(C6A), and **(E, F)** apo-SOD1(C111S). Spectra are shown for samples collected (A, C, E) before and (B, D, F) after H_2O_2 treatment.

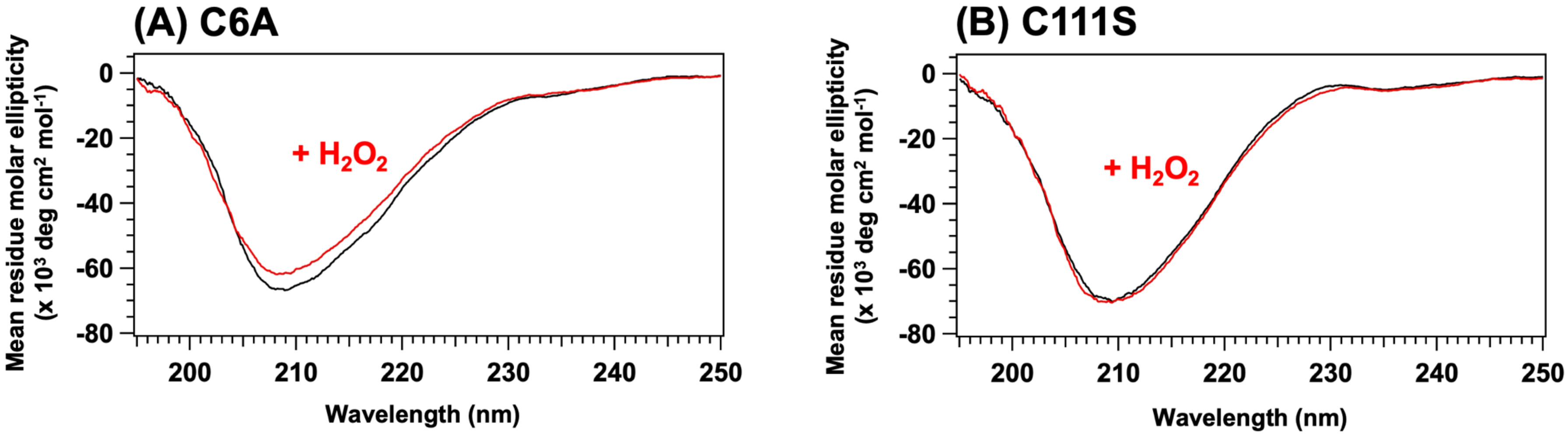

Figure S3 **Both Cys6 and Cys111 are required for oxidative denaturation of SOD1** CD spectra of **(A)** 20 μM apo-SOD1(C6A) and **(B)** 20 μM apo-SOD1(C111S) in 5 mM MOPS/100 mM NaCl (pH 7.0) (black) before and (red) after treatment with 10 mM H_2O_2.

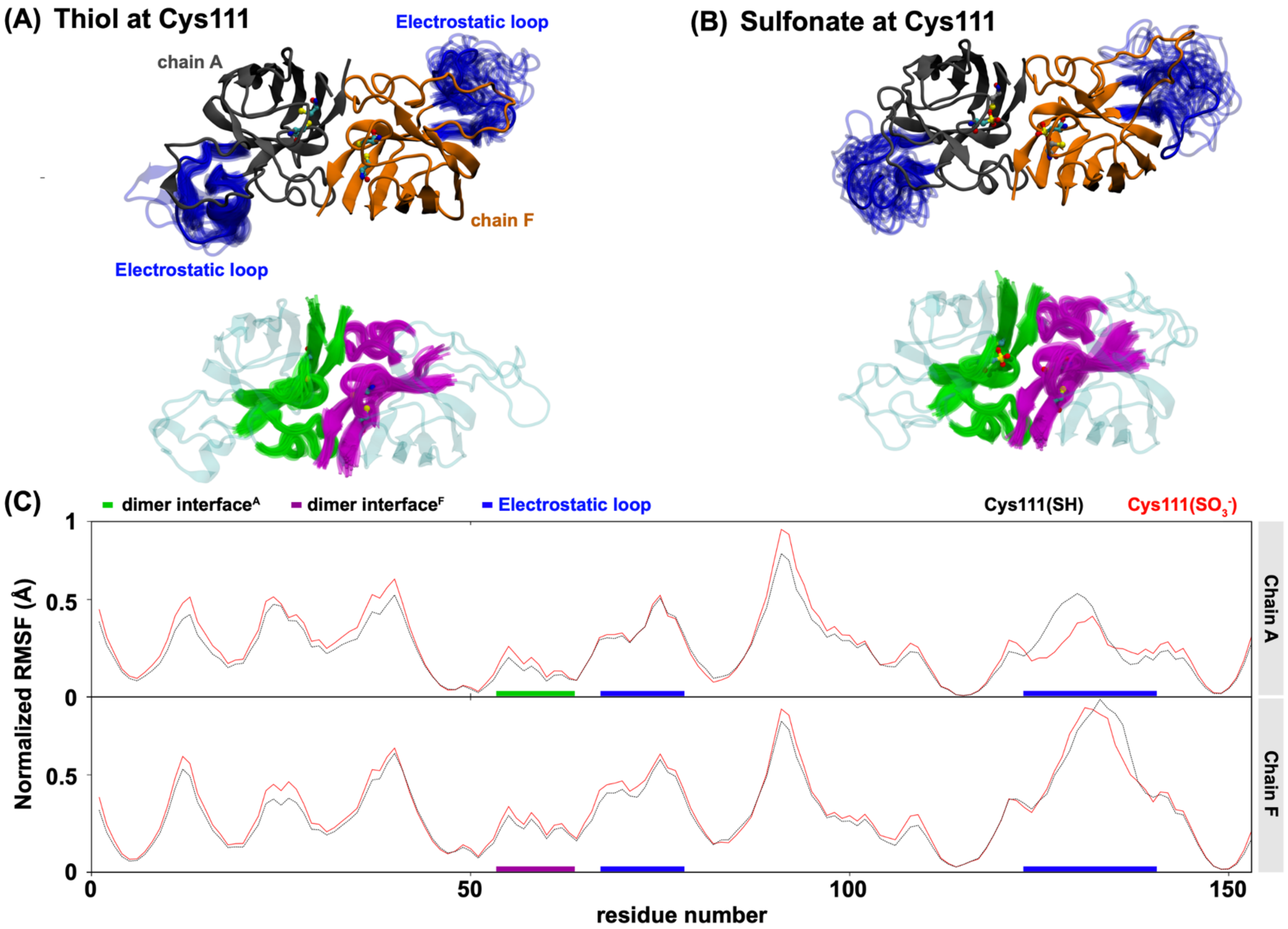

Figure S4 SOD1 conformation is not significantly affected by the oxidation state of Cys111 **(A, B)** The top panels show the three-dimensional structures depicting the motion of the electrostatic loop region (blue) in chain A (grey) and chain F (orange) of SOD1 with Cys111 in the (A) thiol or (B) sulfonate state. In the bottom panels, the structural fluctuations at the dimeric interface are highlighted (light green and purple in chain A and F, respectively). **(C)** Normalized RMSF analysis calculated from the last 200 ns of the MD trajectory compares the structural fluctuations of SOD1 between the (black) thiol and (red) sulfonate state at Cys111. The regions highlighted in (A, B) are indicated with matching color.

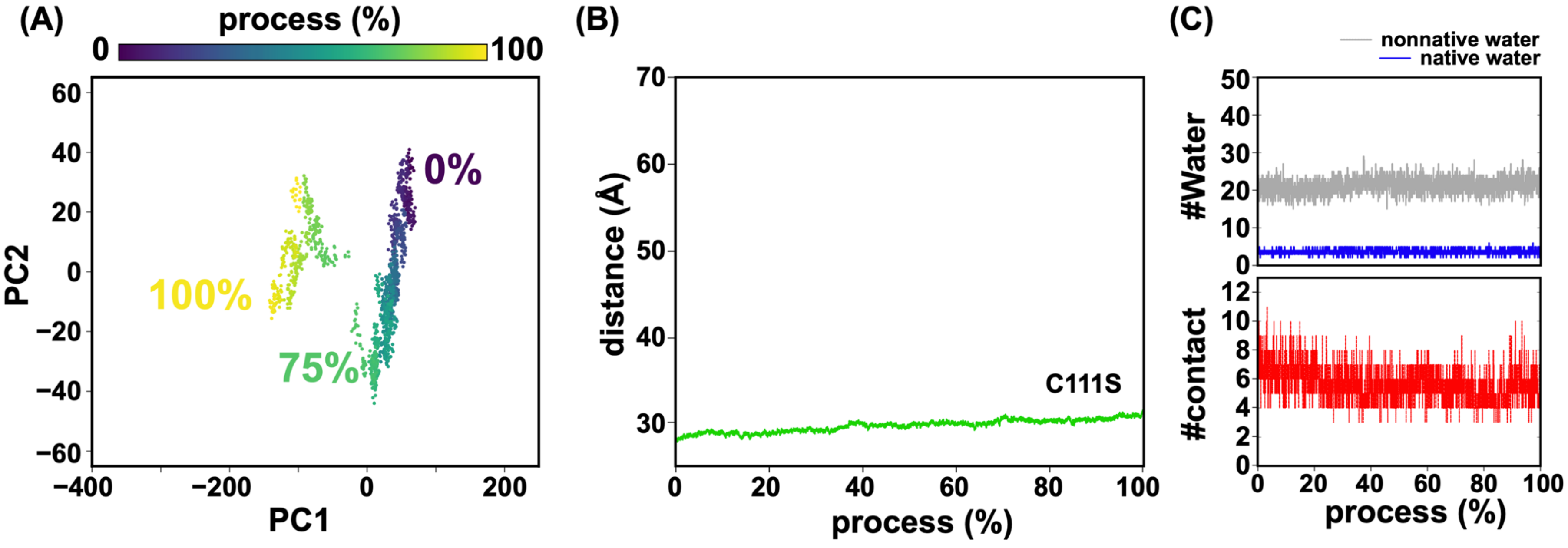

Figure S5 **Dissociation analysis of dimeric SOD1** **(A)** Scatter plot depicting protein motion of SOD1 in the sulfonate state at Cys111 during the dissociation process derived from PCA analysis. **(B)** The distance profile during the dissociation process is shown for SOD1 with C111S substitution. **(C)** The number of water molecules (#Water) between chain A and F of SOD1 with C111S substitution was monitored throughout the simulation, distinguishing between native (blue line) and non-native water (gray line), alongside changes in atomic contacts (#contact).

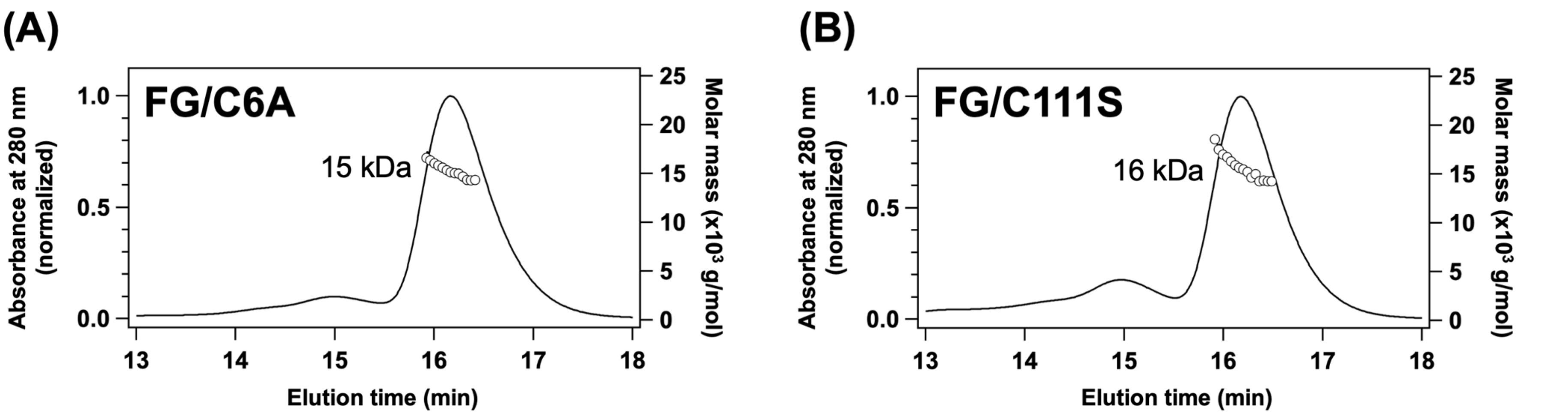

Figure S6 Monomerization of SOD1 induced by F50E/G51E substitutions Gel filtration chromatograms of **(A)** 20 μM apo-SOD1(C6A/FG) and **(B)** 20 μM apo-SOD1(C111S/FG) in the MN buffer, monitored at 280 nm. Chromatograms are normalized for comparison (solid lines, left axis), with molecular masses estimated by MALS (circles, right axis).

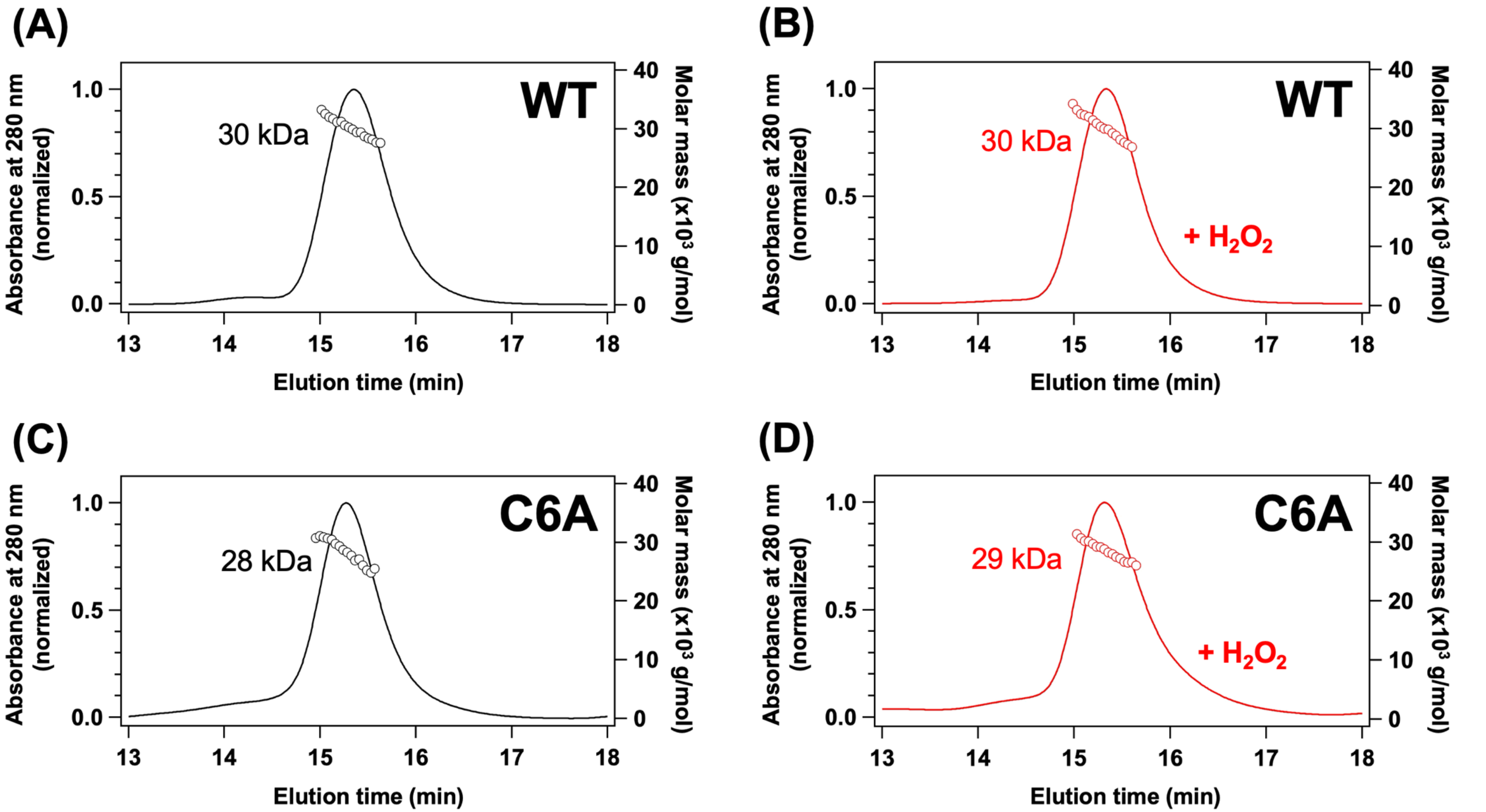

Figure S7 Minimal impact of oxidation on the homodimeric configuration of Zn^{2+}-bound SOD1 Gel filtration chromatograms of **(A, B)** 20 μM SOD1(WT) and **(C, D)** 20 μM SOD1(C6A) in the presence of equimolar $ZnSO_4$ in the MN buffer, monitored at 280 nm. Chromatograms are shown for samples (A, C) before and (B, D) after treatment with H_2O_2. Solid lines represent normalized chromatograms for comparison (left axis), with estimated molecular masses indicated by circles based on MALS analysis (right axis).

Supplemental Movie

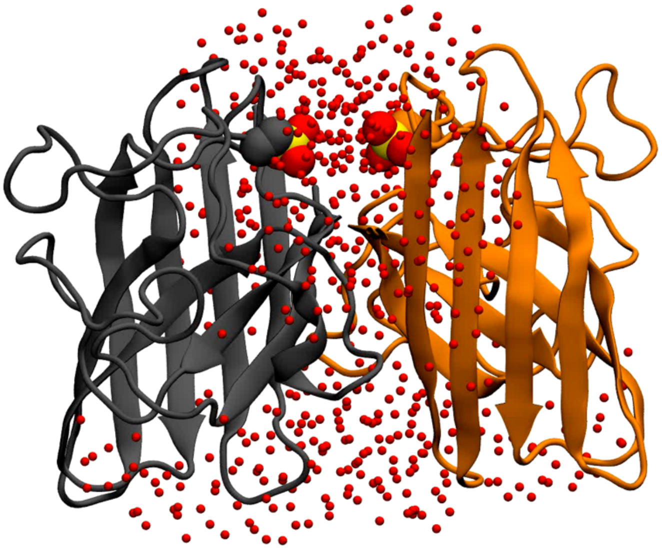

Movie S1 Dimer dissociation process of SOD1 with sulfonated Cys111 based on the LB-PaCS-MD simulation. The two subunits of SOD1 are shown in black and orange for chains A and F, respectively. The side chain of Cys111, in which the thiol group is oxidized to a sulfonate, is displayed as space-filling models. The oxygen atom of water molecules within 6 Å of Cys111 are shown as red spheres.

Supplemental Experimental Procedures

Electrophoresis – SDS-PAGE was performed using a 12.5 or 15% polyacrylamide gel with a 5% polyacrylamide stacking gel. For the electrophoresis in reducing conditions, protein samples were mixed with the Laemmli sample buffer containing β-mercaptoethanol (β-ME). For non-reducing SDS-PAGE, protein samples were first incubated with 100 mM iodoacetamide (IA) in the presence of 2% SDS at 37°C for an hour and then mixed with the Laemmli sample buffer without any reductants. In both reducing and non-reducing conditions, the samples were boiled at 100°C for 5 min prior to electrophoresis. Gels were stained with Coomassie Brilliant Blue R-250 and imaged using a transmission mode scanner.

For experiments involving the modification of SOD1 proteins with maleimide-PEG (mPEG-MAL, 5,000 Da, NANOCS), SOD1 proteins (20 µM) were incubated with 0.1 mg/mL mPEG-MAL in the MN buffer at 37°C for 30 min. The reaction was quenched by adding β-mercaptoethanol (β-ME) to a final concentration of 10% (v/v), and the samples were then mixed with the Laemmli sample buffer and analyzed by SDS-PAGE using 15% polyacrylamide gels.

Hydrophobicity evaluation of SOD1 based on SYPRO^{TM} Orange fluorescence – SOD1 samples at a final concentration of 20 µM in the MN buffer were incubated with SYPRO^{TM} Orange (Invitrogen) at a final concentration of 10x. Fluorescence spectra were recorded from 650 to 500 nm emission wavelengths using a fluorescence spectrophotometer (F-4500, Hitachi) with an excitation wavelength of 491 nm and a scan speed of 240 nm/min.

Circular dichroism spectroscopy – SOD1 samples at a final concentration of 20 µM were prepared in a buffer containing 5 mM MOPS and 100 mM NaCl at pH 7.0. Circular dichroism (CD) spectra were

recorded from 300 to 190 nm using a CD spectrometer (J-1100, JASCO) at 20°C, with a step size of 0.1 nm and a scan speed of 100 nm/min. The final spectrum represents the average of five scans.

Size exclusion chromatography with multi-angle light scattering (SEC-MALS) – SOD1 samples at a final concentration of 20 μM in the MN buffer were loaded on a gel filtration column (Cosmosil-5-Diol-120-II, nacalai tesque) fitted to an HPLC system (Shimadzu), and the absorbance change at 280 nm of the elution was monitored. The molecular size of the protein eluted from the column was determined by multi-angle light scattering using miniDAWN TREOS (WYATT Technology) connected on-line to the HPLC system.

Mass spectrometry – For electrospray ionization mass spectrometry (ESI-MS) under denaturing conditions, samples were desalted using micro-reverse phase chromatography with ZipTip C4 (Millipore, Billerica, MA). The tip was equilibrated with 0.1% formic acid. Samples, diluted in 0.1% formic acid, were loaded on the tip. After washing the tip with 0.1% formic acid, proteins were eluted with 50% acetonitrile containing 0.1% formic acid. For ESI-MS under native conditions (native MS), the buffer of the SOD1 samples was exchanged to 200 mM ammonium acetate (pH 6.8), which had been demetallated by treatment with Chelex 100 Chelating Resin (Bio-Rad, Hercules, CA). The SOD1 samples, at a final concentration of 10 μM, were then mixed in equal volume with 200 mM ammonium acetate (also treated with Chelex resin) supplemented with EDTA at a final concentration of 20 μM. This mixture was used for native MS measurements.

Mass spectral data were acquired using a SYNAPT G2 HDMS instrument (Waters, Milford, MA) equipped with a nano-ESI source. Samples were loaded into platinum-coated borosilicate capillaries with a 1-μm inner diameter (HUMANIX, Hiroshima, Japan). For denaturing conditions, the ESI-MS parameters were set as follows: capillary voltage was set to 0.6 kV; the source temperature was held to 70°C; the sampling cone voltage was set at 40 V; an argon gas flow rate was set at 1.5

mL/min; and the backing pressure in the source region was 2.2–2.3 mbar. For native conditions, the parameters were set as follows: the capillary voltage was set to 0.7 kV; the source temperature was held to 70°C; the sampling cone voltage was set at 60 V; an argon gas flow rate was set at 2.0 mL/min; the backing pressure in the source region was 2.2 - 2.3 mbar.

Quantification of thiols by 5,5'-Dithiobis(2-nitrobenzoic acid) (DTNB) – The protein samples (20 μM) were mixed in equal volume with DTNB (200 μM, Thermo Scientific) in a buffer containing 100 mM Tris and 1 mM EDTA (pH 8.0) with 6 M guanidine-HCl followed by incubation at room temperature for 30 min. Absorption spectra were recorded from 700 to 250 nm using a spectrophotometer (UV-1800, Shimadzu). Following the manufacturer's instructions, the concentration of 2-nitro-5-thiobenzoic acid, generated by the quantitative reduction of DTNB with thiol groups, was determined from the absorbance at 412 nm using a molar extinction coefficient of 13,700 $M^{-1}cm^{-1}$, allowing for the quantification of thiol groups in the protein samples.

Comparative analysis on Zn^{2+}-affinity of SOD1 variants – SOD1 in the apo form (20 μM) was mixed with an equimolar amount of $ZnSO_4$, incubated at 37°C for an hour, mixed in equal volume with ZnAF-2 (20 μM) in the MN buffer. After incubation at 37°C, fluorescence spectra were recorded from 800 to 500 nm using a fluorescence spectrophotometer (F-4500, Hitachi) with an excitation wavelength of 492 nm and a scan speed of 240 nm/min.

Cell viability assay – SOD1 samples (10 - 200 μM) were mixed in equal volume with the MN buffer containing 0.06 mg/mL catalase and 400 μM $ZnSO_4$. SH-SY5Y (EC94030304-F0, ECACC, London, UK) cells were seeded at a density of 3,000 cells/well in 96-well plates and cultured in 100 μL DMEM (10% FBS) for 24 h. SH-SY5Y cells were differentiated in 100 μL DMEM (1% FBS) containing 10 μM retinoic acid (RA) for 7 days, and then cultured in the 90 μL culture medium mixed with 10 μL of the samples with or without 10 μM RA. We confirmed that differentiated SH-SY-5Y cells in the

conditions as described above is not affected by Zn^{2+} ion up to 30 μM. Apo-SOD1 can bind one Zn^{2+} ion per subunit, thus concentration of Zn^{2+} ion in culture medium becomes 20 μM or less and it does not affect cell viability. Cell viability was measured using the Alamar Blue reagent (Thermo Fisher Scientific). The 10 μL Alamar Blue reagent was added to the culture medium, which was subsequently incubated for 4 h. Absorbance was measured at 570 and 600 nm using a Synergy H1 Hybrid multi-mode microplate reader (BioTek, Winooski, VT, USA).

Amino acid analysis – Protein samples were hydrolyzed in constant-boiling HCl at 110°C for 24 h under reduced pressure. Hydrolysates were analyzed using an automated amino acid analyzer (L-8900, Hitachi) equipped with a cation-exchange column (#2620MPH, 4.6 mm x 80 mm) and an ammonia filter column (#2650L, 4.6 mm x 40 mm). Amino acids were separated by ion-exchange chromatography and detected by post-column reaction with ninhydrin (135°C) at 570 nm and 440 nm.

Molecular dynamics (MD) simulation – The crystal structure of dimeric human SOD1 (PDB ID: 2C9V) was used as the starting point for this study, with bound copper and zinc ions, as well as water molecules, removed. Protonation states for each amino acid were assigned at pH 7.0 using PDB2PQR, and two systems were prepared by mutating Cys111 on both chains A and F, resulting in SOD1 (Cys-SH) and oxidized SOD1 (Cys-SO_3^-). For the oxidized SOD1, topology and force field parameter file for the chemical group (SO_3^-) covalently bound to Cys111 were generated using Gaussian16, with the MCPB.py script and the B3LYP/6-31G(d,p) basis set, following previously described procedures (1). Each protein system was solvated in a TIP3P water cubic box with a minimum distance of 12 Å from the protein surface and neutralized using Na^+ via the tLEaP program in AMBER22 (2). Molecular dynamics (MD) simulations were performed using the AMBER22 package under periodic boundary conditions (PBCs), beginning with energy minimization using steepest descent and conjugate gradient methods, followed by structural relaxation with harmonic

restraints using the SANDER program. The systems were gradually heated to 310 K for 0.01 μs-MD trajectory, equilibrated for 0.11 ns, and then subjected to production simulations for 1 μs-MD trajectories with three independent replicates per system. The average deviation of atomic positions from their mean positions over time for each system was analyzed based on root-mean-square-fluctuation (RMSF). Root mean square deviation (RMSD) analysis of the loop region (residues 121-142) and the number of water contacts at Cys6 were performed using the CPPTRAJ module in AMBER 22. The last 200 ns-MD trajectory was used to analyze for RMSF and the binding energy between chains A and F based on MM/PB(GB)SA method. Additionally, the SOD1 dimer dissociation was evaluated by the LB-PaCS-MD technique (3; 4), which was the enhanced sampling method. The principal component analysis (PCA) was used to investigate the protein motion along the dissociation.

1. Sinsulpsiri S, Nishii Y, Xu-Xu QF, Miura M, Wilasluck P, Salamteh K, Deetanya P, Wangkanont K, Suroengrit A, Boonyasuppayakorn S, Duan L, Harada R, Hengphasatporn K, Shigeta Y, Shi L, Maitarad P, Rungrotmongkol T (2025) Unveiling the antiviral inhibitory activity of ebselen and ebsulfur derivatives on SARS-CoV-2 using machine learning-based QSAR, LB-PaCS-MD, and experimental assay. Sci Rep 15:6956.
2. Case DA, Belfon K, Ben-Shalom I-Y, Brozell SR, Cerutti DS, Cheatham TE, Cruzeiro VWD, Darden TA, Duke RE, Giambasu G, Gilson MK, Gohlke H, Goetz AW, Harris R, Izadi S, Izmailov SA, Kasavajhala K, Kovalenko A, Krasny R, Kurtzman T, Lee T-S, LeGrand S, Li P, Lin C, Liu J, Luchko T, Machado MR, Man V, Merz KM, Miao Y, Mikhailovskii O, Monard G, Nguyen H, Onufriev A, Pan F, Pantano S, Qi R, Rahnamoun A, Roe DR, Roitberg A, Sagui C, Schott-Verdugo S, Shajan A, Shen J, Simmerling CL, Skrynnikov NR, Smith JS, Swails JM, Walker RC, Wang J, Wei H, Wolf RM, Wu X, Xiong Y, Xue L, York DM, Zhao Y, Zubatyuk R. 2022. Amber 2022, University of California, San Francisco.
3. Aida H, Shigeta Y, Harada R (2022) Ligand Binding Path Sampling Based on Parallel Cascade Selection Molecular Dynamics: LB-PaCS-MD. Materials (Basel) 15.
4. Hengphasatporn K, Harada R, Wilasluck P, Deetanya P, Sukandar ER, Chavasiri W, Suroengrit A, Boonyasuppayakorn S, Rungrotmongkol T, Wangkanont K, Shigeta Y (2022) Promising SARS-CoV-2 main protease inhibitor ligand-binding modes evaluated using LB-PaCS-MD/FMO. Sci Rep 12:17984.
